# A new genus of freshwater crab from Vietnam, with comments on the allied genera *Vietorientalia* Đặng, 2012, and *Hainanpotamon* Dai, 1995 (Decapoda, Brachyura, Potamidae)

**DOI:** 10.3897/zookeys.1294.199712

**Published:** 2026-10-05

**Authors:** Zhi Wan Tan, Van Tri Ngo, Boyang Shi

**Affiliations:** 1 Department of Biological Sciences, Faculty of Science, National University of Singapore, Singapore 117558, Singapore Department of Biological Sciences, Faculty of Science, National University of Singapore Singapore Singapore; 2 Saigon Institute of Applied Sciences, 52 Bau Cat 6 Street, Tan Binh Ward, Ho Chi Minh City, Vietnam Saigon Institute of Applied Sciences Ho Chi Minh City Vietnam; 3 Ministry of Education Key Laboratory for Ecology of Tropical Islands, Key Laboratory of Tropical Animal and Plant Ecology of Hainan Province, College of Life Sciences, Hainan Normal University, Haikou, China Ministry of Education Key Laboratory for Ecology of Tropical Islands, Key Laboratory of Tropical Animal and Plant Ecology of Hainan Province, College of Life Sciences, Hainan Normal University Haikou China; 4 Jiangsu Key Laboratory for Biodiversity and Biotechnology, College of Life Sciences, Nanjing Normal University, Nanjing, 210023, China Jiangsu Key Laboratory for Biodiversity and Biotechnology, College of Life Sciences, Nanjing Normal University Nanjing China

**Keywords:** 16s, COI, Crustacea, Hainan Island, new combination, Potamiscinae, taxonomy, Vietnam

## Abstract

The nomenclatural history and taxonomic problems associated with two poorly known Vietnamese potamid freshwater crab species assigned initially to *Hainanpotamon* Dai, 1995, and later to *Vietorientalia* Đặng, 2012, viz., *V. glabra* (Đặng, 1967) and *V. rubra* (Đặng & Trần, 1992), are addressed. Clear morphological and molecular evidence confirms the validity of *Vietorientalia*, and a neotype is herein designated for the type species, *V. glabra*, for which the type material is purportedly lost. Moreover, morphological and molecular evidence suggests that the two species currently assigned to *Vietorientalia* are non-congeneric. Accordingly, a new genus, *Dangmon***gen. nov**., is herein erected to accommodate *V. rubra*.

## Introduction

The freshwater potamid crabs of Vietnam are diverse and include several genera characterised by a globose, highly inflated carapace, viz., *Binhthuanomon* Do, Le & Phan, 2015, *Hainanpotamon* Dai, 1995, *Lacunipotamon* Dai, Song, He, Cao, Xu & Zhong, 1975, *Laevimon* Yeo & Ng, 2005, and *Vietorientalia* Đặng, 2012. Among these genera, the taxonomic histories and relationship between *Hainanpotamon* and *Vietorientalia* are particularly confusing and require clarification. *Vietorientalia* was established by Đặng (2012) for two semi-terrestrial crab species endemic to Vietnam, viz., *Vietorientalia glabra* (Đặng, 1967) (type species) and *V. rubra* (Đặng & Trần, 1992). The generic placements of these two species, however, have been the subject of some confusion and debate (Yeo and Ng 1998, 2005; Yeo and Naruse 2008; Đặng 2012).

*Vietorientalia glabra* (Đặng, 1967) was originally described as a species of Potamon (Geothelphusa) Stimpson, 1858. Đặng (1975) subsequently erected the genus *Orientalia* Đặng, 1975, for Potamon (Geothelphusa) glabra Đặng, 1967 (locality: Hoa Binh, Ha Tay, Bac Thai Provinces, northern Vietnam) and Potamon (Potamon) orientale Parisi, 1916 (locality: Hainan Island, China), with Đặng and Trần (1992) adding two more species from central Vietnam (*O. rubra* and *O. tankiense*), bringing the total number of *Orientalia* species to four. Almost concurrently, Dai (1995), unaware of Đặng’s (1975) establishment of *Orientalia* and inclusion of Potamon (Potamon) orientale Parisi, 1916, established a new genus *Hainanpotamon* for potamid species on Hainan Island, with Potamon (Potamon) orientale Parisi, 1916, as the type species. The name *Orientalia*, however, was shown by Yeo and Ng (1998) to be preoccupied by *Orientalia* Radoman, 1972, a genus of freshwater snails from the Balkans [itself now replaced by *Radomaniola* Szarowska, 2006, after it was found also to be invalid as a junior homonym of *Orientalia* Bykova, 1947, a foraminiferan genus (Szarowska 2006)]. To resolve the nomenclatural problem caused by the homonymy, Yeo and Ng (1998) regarded *Orientalia* Đặng, 1975, as a junior subjective synonym of *Hainanpotamon* Dai, 1995, and transferred all *Orientalia* crab species to the latter genus group, citing the similar external and gonopodal morphology of the species and the priority of the genus name.

In a later study, Yeo and Ng (2005) transferred *Hainanpotamon tankiense* (Đặng & Trần, 1992) to the new genus *Laevimon* Yeo & Ng, 2005 (type species: *L. kottelati* Yeo & Ng, 2005) based on clear differences in the sternal and male gonopod morphology of the two genera. Yeo and Naruse (2008) described three more species of *Hainanpotamon*, namely, *H. daiae* Yeo & Naruse, 2008 (locality: Hainan Island, China), *H. auriculatum* Yeo & Naruse, 2008 (locality: Da Nang Province, Central Vietnam), *H. directum* Yeo & Naruse, 2008 (locality: Nam Noy, Northern Laos), and added the poorly known species Potamon (Geothelphusa) globosum Parisi, 1916, to the genus. However, Ng et al. (2008) listed *Hainanpotamon globosum* (Parisi, 1916) under *Nanhaipotamon* Bott, 1968, without any explanation.

Most recently, while recognising the name *Orientalia* Đặng, 1975, as invalid because of its status as a junior homonym and disagreeing with the placement or retention of *H. rubrum* (Đặng & Trần, 1992) and *H. glabrum* (Đăng, 1967) in *Hainanpotamon* (Yeo and Ng 1998; Yeo and Naruse 2008), Đặng (2012) established the genus *Vietorientalia* as a replacement name for *Orientalia* Đặng, 1975, to accommodate *H. rubrum* (Đặng & Trần, 1992) and *H. glabrum* (Đặng, 1967). Therefore, *Hainanpotamon* is now represented by six species, viz., *H. orientale* (Parisi, 1916), *H. fuchengense* Dai, 1995, *H. helense* Dai, 1995, *H. daiae* Yeo & Naruse, 2008, *H. auriculatum* Yeo & Naruse, 2008, and *H. directum* Yeo & Naruse, 2008.

The validity of the genus *Vietorientalia*, however, still requires clarification. Đặng (2012) provided only a brief diagnosis and arguments supporting the establishment of the genus, without accompanying illustrations or photographs. The descriptions and illustrations in Đặng (1967, 1980) and Đặng and Hồ (2012) are also unclear and do not provide an accurate depiction of the species’ morphology. In their revision of the genus *Hainanpotamon*, Yeo and Naruse (2008) were unable to examine the holotype of either *Vietorientalia glabra* or *V. rubra*, the former of which was reported as lost. As a result, the actual carapace and gonopodal morphology of the two species, as well as the validity of the genus, have remained uncertain. Recently, the second author collected specimens of both species from their type localities, providing an opportunity to reassess the validity the genus *Vietorientalia* Đặng, 2012, and clarify its relationship with *Hainanpotamon* Dai, 1995.

## Material and methods

The specimens are preserved in 75% ethanol. They were subsequently deposited in the Zoological Reference Collection of the Lee Kong Chian Natural History Museum, National University of Singapore (ZRC), and Jiangsu Key Laboratory for Biodiversity and Biotechnology, Nanjing Normal University (NNU). The morphological terminology used in the descriptive accounts follows Ng (1988), with subsequent updates based on Guinot et al. (2013) and Davie et al. (2015). Measurements were taken of the carapace maximum width (**CW**) and length along the midline (**CL**), respectively. All measurements were taken in millimetres (mm) using dial callipers. Specimens were examined using Leica M80 and M205c stereomicroscopes and illustrations were made using the mounted drawing tubes. Photographs of specimens were taken using an Olympus OM-D E-M1 Mark III mirrorless interchangeable lens camera with a M.Zuiko Digital ED 60 mm F2.8 lens. Figures were edited and assembled using Adobe Photoshop Lightroom and Adobe Photoshop. The following abbreviations are used: **asl**, above sea level; **G1**, male first gonopod; **G2**, male second gonopod.

To determine the systematic position and taxonomic status of the new taxon, four fragments from 17 individuals were used to reconstruct phylogenetic relationships. The dataset included 13 potamiscines as the ingroup and two potamines as the outgroup, based on two mitochondrial sequences [cytochrome c oxidase subunit I (COI) and 16S ribosomal RNA (16S)] and two nuclear sequences [28S ribosomal DNA (28S) and histone H3 (H3)]. Total genomic DNA were extracted from the gill or muscle tissue from the ambulatory legs using the Tiangen universal DNA purification kit, following the manufacturer’s protocol. The quality of the samples was assessed using 1% agarose gels and a NanoDrop 2000 spectrophotometer (Thermo Scientific). Extracted DNA was stored in a refrigerator until required for polymerase chain reaction (PCR) amplification. All primers used are listed in Suppl. material 1. PCRs were performed in a total volume of 25 µL, containing 25 ng of genomic DNA, 1.5 mM MgCl_2_, 0.5 mM dNTPs, 0.6 µM primers, and 0.75 units of Taq DNA polymerase. PCR conditions were as follows: an initial denaturation at 94 °C for 3 min; 35 cycles of denaturation at 94 °C for 30 s, annealing at the temperatures listed in Suppl. material 1 for 40 s, and extension at 72 °C for 50 s; followed by a final extension at 72 °C for 7 min. PCR products were gel-purified and sequenced using an ABI 3730XL automatic sequencer. Both strands were sequenced using the same primers as for PCR amplification. The sequences have been deposited in GenBank (see Suppl. material 2 for accession numbers).

All sequences were aligned using MAFFT v. 7.310 with the G-INS-i strategy (Katoh and Standley 2013). All alignments were trimmed with Gblocks v. 0.91b (Castresana 2000) and concatenated using custom Python scripts. According to the Akaike information criterion (AIC) and Bayesian information criterion (BIC), ModelFinder determined GTR + I + G to be the best-fitting model (Kalyaanamoorthy et al. 2017). The Bayesian inference (BI) analysis was performed using MrBayes v. 3.2.7a (Ronquist et al. 2012). Metropolis-coupled Markov chain Monte Carlo (MCMC) analyses were run with one cold chain and three heated chains for 1 × 10^7^ generations, with sampling every 1,000 generations. Four independent MCMC runs were performed, and 1,000 trees were discarded from each run as burn-in. Confidence in the topology was assessed using posterior probability (Huelsenbeck and Ronquist 2001). Effective sample size (ESS) values were checked using Tracer v. 1.7 (Rambaut et al. 2018) to ensure that all values exceeded 200. The maximum likelihood (ML) analysis was performed using IQ-TREE v. 2 (Minh et al. 2020), with 1,000 bootstrap replicates. The phylogenetic trees were visualised using iTOL v. 5 (Letunic and Bork 2021).

## Taxonomy

### Family Potamidae Ortmann, 1896

**Subfamily Potamiscinae Bott, 1970 (sensu Yeo and Ng 2004)**

#### Vietorientalia

Taxon classificationAnimaliaDecapodaPotamidae

Đặng, 2012

B158A917-CD19-583E-904B-9B84EAC83039

Figs 1, 2, 3, 4

Orientalia Đăng, 1975: 75.—Đặng and Trần 1992: 17 (part) [invalidated by senior homonym Orientalia Bykova, 1947 (Foraminifera: Lituolida)].
Vietorientalia Đặng, 2012: 306.—Đặng and Hồ 2012: 188.—Ng et al. 2023: 2–4.

##### Type species.

Potamon (Geothelphusa) glabra Đặng, 1967, by original designation. Gender of genus feminine.

##### Diagnosis.

**Figure 1.**
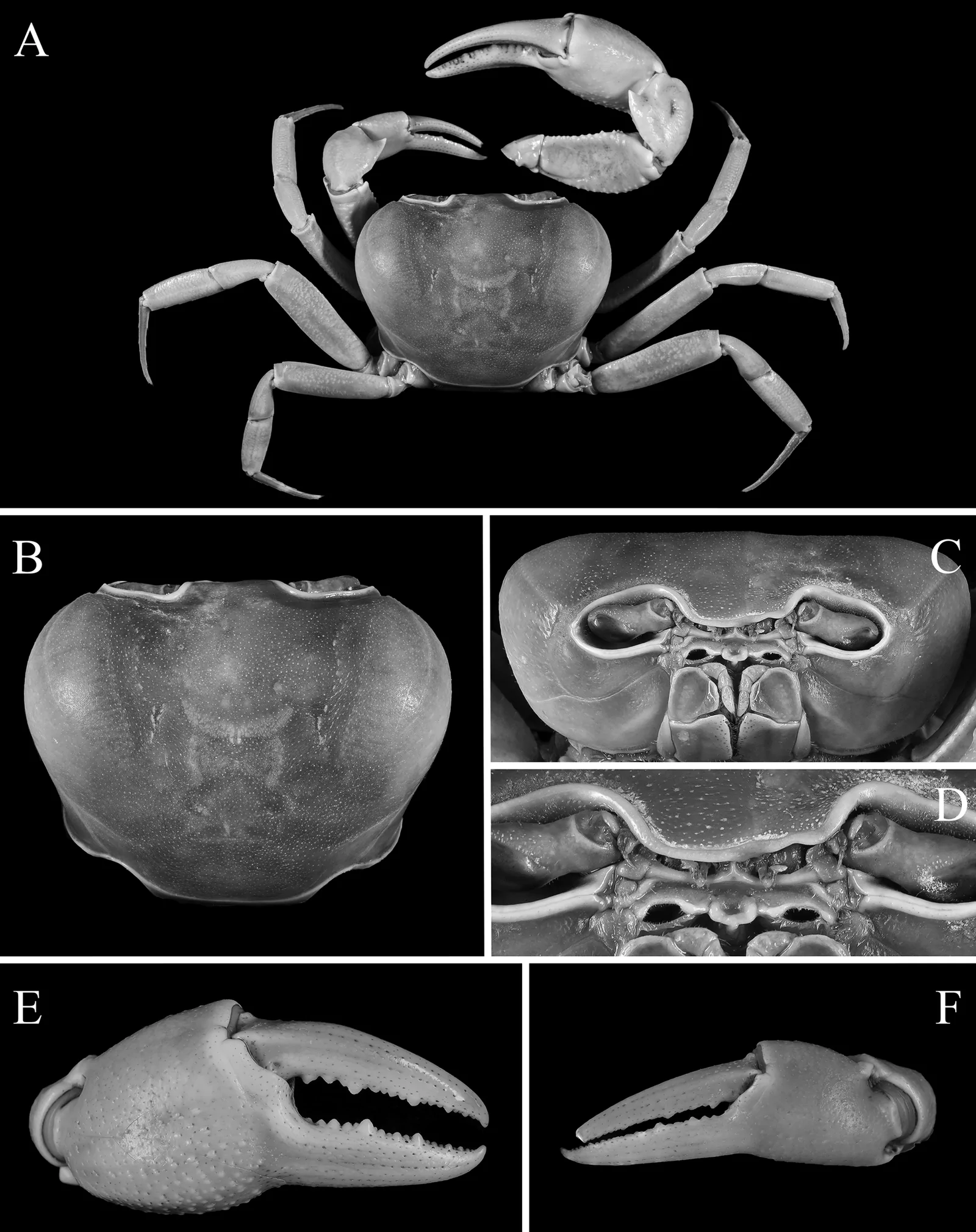
*Vietorientalia glabra* (Đặng, 1967), neotype, male (46.1 × 35.4 mm) (ZRC 2024.0291), Phú Thọ province, Vietnam. **A**. Overall dorsal habitus; **B**. Dorsal view of cephalothorax; **C**. Frontal view of cephalothorax; **D**. Antennae, antennules and epistome; **E**. Major chela (right); **F**. Minor chela (left).

**Figure 2.**
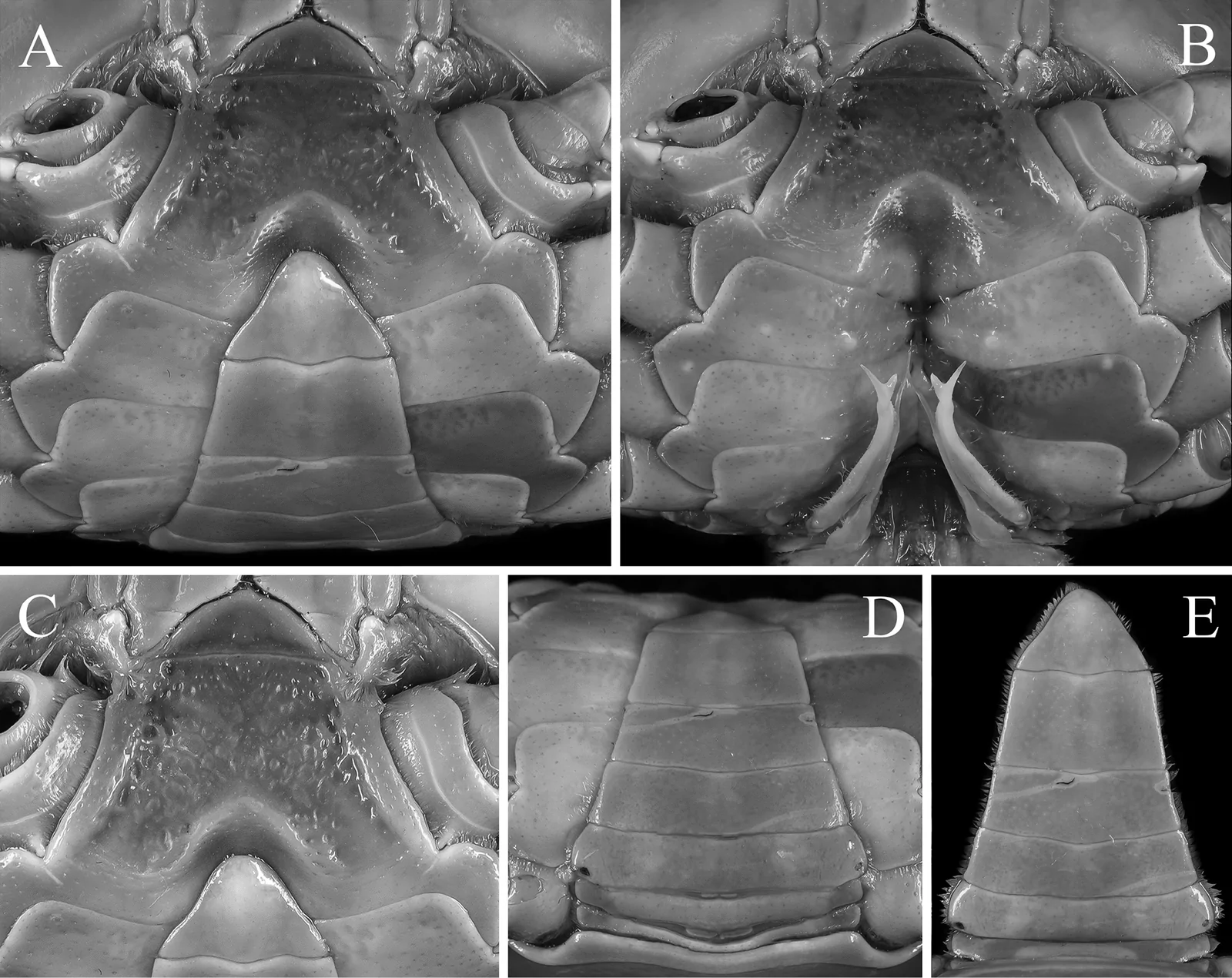
*Vietorientalia glabra* (Đặng, 1967), neotype, male (46.1 × 35.4 mm) (ZRC 2024.0291), Phú Thọ province, Vietnam. **A**. Thoracic sternites, pleon and telson; **B**. Sternopleonal cavity with G1 and G2 in-situ; **C**. Anterior thoracic sternites 1–4; **D**. Posterior thoracic sternum and pleon; **E**. Pleon.

**Figure 3.**
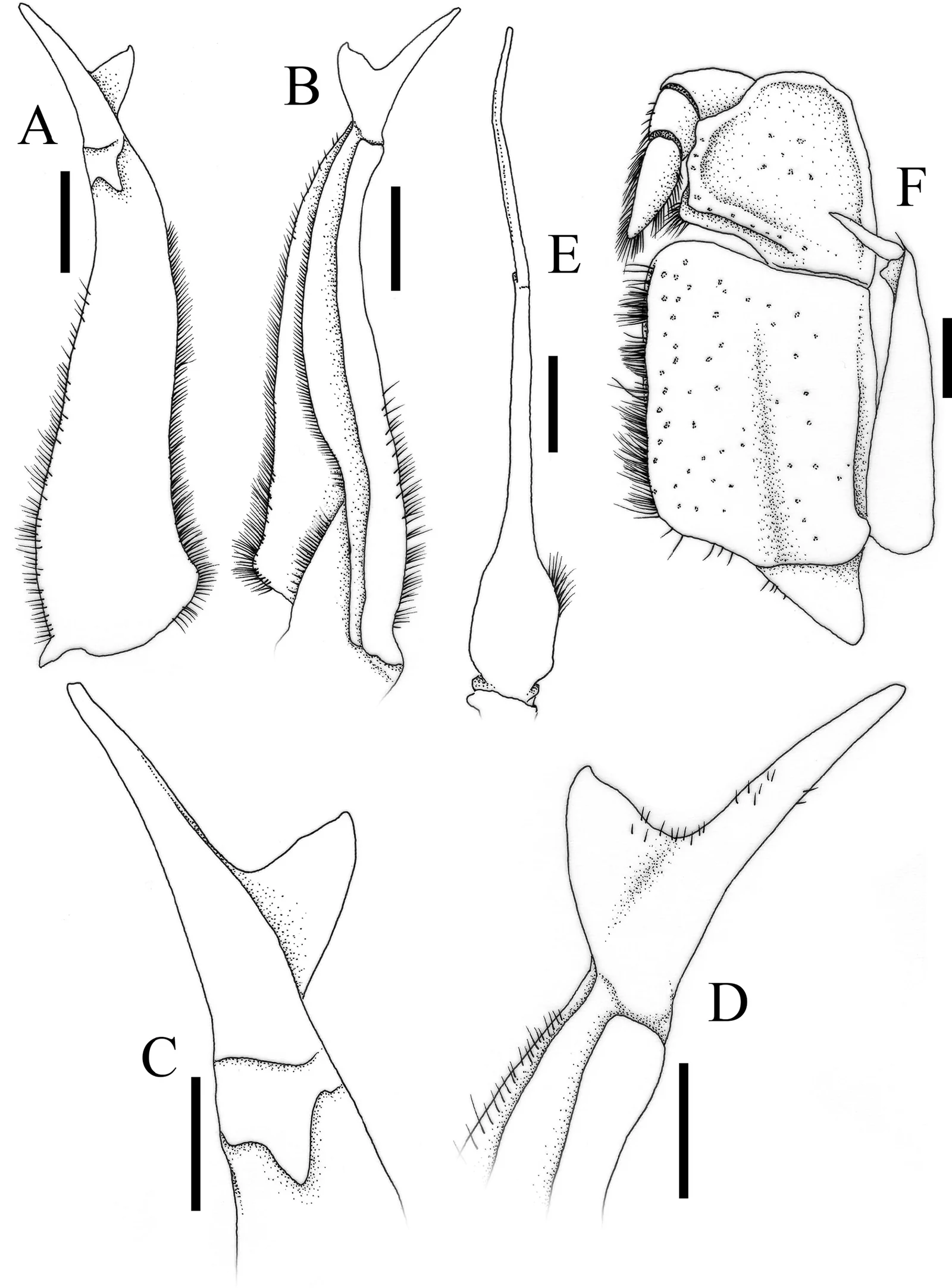
*Vietorientalia glabra* (Đặng, 1967), neotype, male (46.1 × 35.4 mm) (ZRC 2024.0291), Phú Thọ province, Vietnam. **A**. Left G1 dorsal view; **B**. Left G1 ventral view; **C**. Left G1 terminal article dorsal view; **D**. Left G1 terminal article ventral view; **E**. Left G2; **F**. Left third maxilliped. Scale bars: 2 mm **(A, B, E, F**); 1 mm (**C, D**).

**Figure 4.**
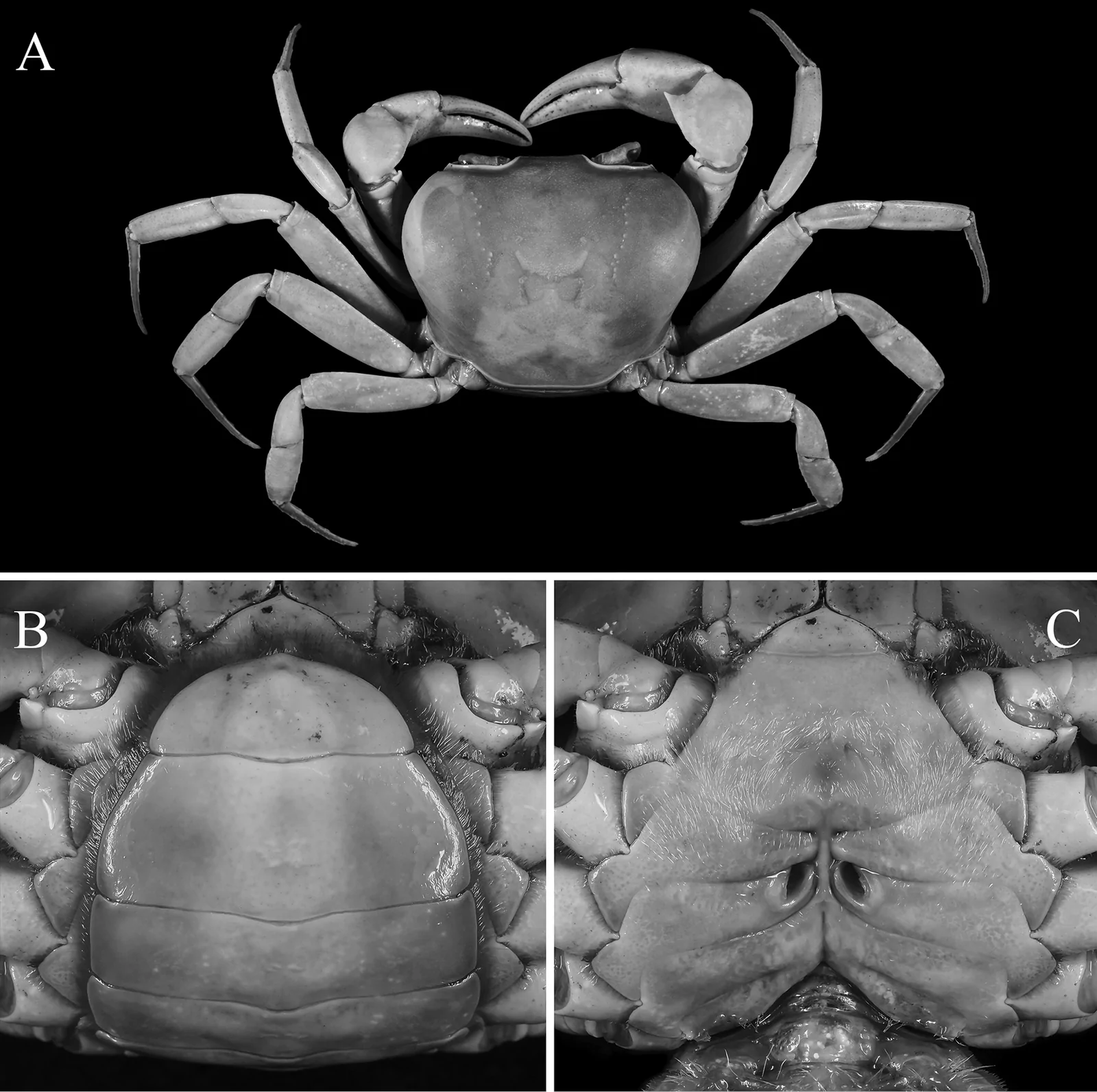
*Vietorientalia glabra* (Đặng, 1967), female (49.1 × 33.2 mm) (ZRC 2022.784), Phú Thọ province, Vietnam. **A**. Overall dorsal habitus; **B**. Pleon and telson; **C**. Sternopleonal cavity and vulvae.

Carapace (Fig. 1A–C) slightly broader than long, high; dorsal surface strongly convex longitudinally, weakly convex transversely, branchial regions gently inflated, smooth, grooves indistinct, regions not well defined; epigastric cristae almost indiscernible, slightly anterior and confluent with postorbital cristae; postorbital cristae very weak, rounded; external orbital tooth triangular, low but discernible, separated from epibranchial tooth by weak, broad cleft; epibranchial tooth indistinct; anterolateral margin distinctly convex, completely smooth. Third maxilliped (Fig. 3F) with short flagellum, subequal to half merus width. Chelipeds (Fig. 1E, F) in adult males unequal, fingers not gaping strongly; ambulatory legs (Figs 1A, 4A) elongate, with very long, slender dactyli. Thoracic sternites 3 and 4 (Fig. 2A, C) with clefts demarcating suture; male sternopleonal cavity barely reaching imaginary line joining medial part of cheliped coxae. G1 (Fig. 3A–D) long, slender, gently sinuous; terminal article slender, subconical, gently curving outwards, with blunt tip and very pronounced dorsal flap in proximal part.

##### Remarks.

Đặng (2012) established *Vietorientalia* to accommodate two species from northern Vietnam, viz., *V. glabra* (Đặng, 1967) [type species] and *V. rubra* (Đặng & Trần, 1992), which were originally placed in the genus *Orientalia* (see Đặng 1975; Đặng and Trần 1992) and subsequently assigned to *Hainanpotamon* by Yeo and Ng (1998) and Yeo and Naruse (2008). During the assessment by Yeo and Naruse (2008), the authors were unable to examine any specimens of *V. glabra*, stating that the holotype was probably lost and relying instead on diagrams from Đặng (1980). In addition, for *V. rubra*, the authors were able to examine a female paratype, as the male holotype was not available for examination, and therefore relied again on diagrams from Đặng and Trần (1992) for the male morphology. The fresh specimens were collected from the type localities of both species (Hoa Binh – *V. glabra*; Thanh Hoa – *V. rubra*) and, based on our examination of the morphology of the G1, pleon, frontal region and orbits, clearly represent the species described by Đặng (1967) and Đặng and Trần (1992), respectively. Based on our morphological and molecular assessment, we also concur that *Vietorientalia* is a valid genus distinct from *Hainanpotamon*. The two species of *Vietorientalia* sensu Đặng (2012), however, display morphological differences, and the results from molecular analysis also support the conclusion that they are not congeneric (see remarks for *Dangmon* gen. nov.). As such, the genus *Vietorientalia* is here restricted to its type species, *V. glabra*, while *V. rubra* is assigned to a new genus, *Dangmon* gen. nov. (see later). Differences between *Vietorientalia* and *Dangmon* gen. nov. are discussed in the genus remarks for the latter.

*Vietorientalia* can be easily distinguished from *Hainanpotamon* sensu stricto (*H. orientale*, *H. fuchengense*, *H. helense*, and *H. daiae*) by the following morphological features: carapace weakly convex transversely, branchial regions inflated (Fig. 1C) (vs carapace appears more strongly convex transversely, branchial regions not prominently inflated; cf. Yeo and Naruse 2008: figs 1B, 4B); sub-hepatic, pterygostomial, and sub-branchial regions smooth (Fig. 1C) (vs sub-hepatic, pterygostomial, and sub-branchial regions being rugose to granulose, Fig. 11; cf. Yeo and Naruse 2008: figs 1B, 4B); postorbital cristae very weak, almost indiscernible, or only visible at lateral edges (Figs 1A, 1B, 4A) (vs postorbital cristae being distinct and discernible across the length of postorbital margins, Fig. 10; cf. Yeo and Naruse 2008: figs 1A, 4A); anterolateral margins entire, smooth (Figs 1A, 1B, 4A) (vs anterolateral margins lined with granules and appearing serrated, Fig. 10; cf. Yeo and Naruse 2008: figs 1B, 4A, B); ambulatory legs more elongate, with distinctly elongate and slender dactyli (Figs 1A, 4A) (vs ambulatory legs being less elongate and stouter, with stout dactyli, Fig. 10; cf. Yeo and Naruse 2008: figs 1A, 4A); and G1 terminal article very slender, curving outwards with a distinct and high dorsal flap on the proximal part (Fig. 3C, D) (vs G1 terminal article being broader, curving gently upwards, with a relatively low dorsal flap on the proximal part, Fig. 13A, B, D, E, G, H, J, K; cf. Yeo and Naruse 2008: figs 3C, D, 5E, F).

##### Geographical distribution.

Phú Thọ province, northern Vietnam.

#### Vietorientalia glabra

Taxon classificationAnimaliaDecapodaPotamidae

(Đặng, 1967)

29A2F88C-321E-5D4E-AAB4-F9B5B884BF8C

Figs 1, 2, 3, 4

Potamon (Geothelphusa) glabra Đặng, 1967: 159, fig. 6.
Orientalia glabra —Đặng 1975: 75; 1980: 425, fig. 242; 1992: 317.
Hainanpotamon glabrum —Yeo and Ng 1998: 358.—Yeo and Naruse 2008: 1150.—Ng et al. 2008: 162.—Do 2014: 111.
Hainanpotamon glabra —Yeo and Ng 1999: 641.
Vietorientalia glabra —Đặng 2012: 306.—Đặng and Hồ 2012: 190, fig. 66.

##### Material examined.

***Neotype***: Vietnam • male (46.1 × 35.4 mm); Lạc Sơn–Ngổ Luông Natural Reserve, Ngọc Sơn commune, Phú Thọ province, northern Vietnam, 20.43°N, 105.37°E; 16 May 2022; local villagers, through Ngo Van Tri leg.; ZRC 2024.0291.

***Non-type specimen***: Vietnam • 1 female (49.1 × 33.2 mm); same collection data as for neotype; ZRC 2022.784.

##### Description.

**Neotype male**. Carapace (Fig. 1A, B) transversely sub-ovate, wider than long (CW/CL ratio: 1.30), high; dorsal surface strongly convex longitudinally, weakly convex transversely due to gently inflated branchial regions, completely smooth but finely punctate. Frontal regions finely punctate, orbital regions very narrow, smooth; frontal margin strongly deflexed downwards, gently convex in frontal view. Suborbital, pterygostomial, subhepatic, sub-branchial regions smooth and sparsely punctate. Epigastric cristae smooth, almost indiscernible, appearing as two low humps; postorbital cristae smooth, very low, confluent with epibranchial tooth and anterolateral margins. H-shaped median groove, cervical grooves indiscernible. External orbital tooth very weakly produced, blunt, broadly triangular, outer margin gently convex, slightly more than length of inner margin, demarcated from rest of anterolateral margin by broad, shallow cleft. Epibranchial tooth very weak, appearing as a nodule or rounded angle. Anterolateral margins strongly convex, subcristate. Posterolateral margins strongly converging towards posterior carapace margin.

Orbits (Fig. 1C) large, ovate; eye partially filling orbital space; ocular peduncle short; cornea large, ovate, pigmented. Supraorbital margin almost straight, cristate, smooth. Suborbital margin concave, cristate, smooth, continuous with supraorbital margin. Antennae very short, barely produced; antennules short, folding transversely in subrectangular fossa. Posterior margin of epistome (Fig. 1D) with distinct, squarish, broad median tooth, inner margins convex, outer margins concave.

Third maxillipeds (Figs 1C, 3F) covering most of buccal cavity when closed; ischium subrectangular, with distinct median groove, surface very sparsely pitted, otherwise smooth; merus subquadrate, nearly as wide as long, surface smooth, margins subcristate; exopod slender, just exceeding anterior edge of ischium, inner subdistal portion produced, with short flagellum not reaching half of merus width.

Chelipeds (Fig. 1A, E, F) strongly unequal, right cheliped larger. basis-ischium anterior margin lined with small rounded granules, otherwise smooth; merus margins lined with low rounded granules; carpus dorsal surface smooth, inner distal angle with large, broad, acute tooth, basal tooth not produced; chelae outer surface granulose. Major chela appearing more robust, proportionally taller and stouter as compared to minor chela. Fingers of major chela, longer than palm, forming small gape when fingers closed; small gape in minor chela; cutting edges of both fingers with variously sized sharp teeth and denticles.

Ambulatory legs (Figs 1A, 4A) relatively long; second pair longest, last pair shortest. Merus long, slender, dorsal surface smooth, dorsal margin entire, without subdistal spine or tooth; carpus smooth, dorsal margin smooth, dorsal surface with very weak sub-median crista; dorsal margin of propodus smooth, dorsal surface with very weak sub-median groove; dactylus very elongate, longer than propodus, distally gently curving, flattened in cross section, margins with sharp spines.

Thoracic sternum (Fig. 2A, C), notably sternites 3, 4, relatively broad, surface strongly pitted. Sternites 1, 2 completely fused forming broadly triangular plate, separated from sternite 3 by distinct, convex suture; sternites 3, 4 fused, only with clefts on lateral edges demarcating suture. Sutures 4/5, 5/6, 6/7, 7/8 medially interrupted; median longitudinal groove on sternites 7, 8. Sternopleonal cavity (Fig. 2B) deep, barely reaching imaginary line joining medial part of cheliped coxae. Tubercle of sternopleonal locking mechanism (press-button) located posteriorly on sternite 5, closer to suture 5/6.

Pleon (Fig. 2E) triangular, all somites, telson free; telson broadly triangular, lateral margins straight; somite 6 trapezoidal, slightly wider than long, lateral margin almost straight; somites 3–5 trapezoidal, gradually decreasing in width, increasing in length; somites 1, 2 (Fig. 2D) subrectangular, very wide, reaching to bases of coxae of fourth ambulatory legs, thoracic sternite 8 not visible when pleon closed.

G1 (Fig. 3A–D) gently sinuous, slender; subterminal article weakly tapering distally, without any subrectangular cleft on upper part of outer margin; terminal article very gently bent outwards, clearly separated from subterminal article, slender, about one-third times length of subterminal article, subconical, very gently curving posteriorly, distal part tapered to blunt tip with distal opening, groove for G2 sub-marginal, with well-produced right-triangular shaped, basal dorsal flap, with apex near middle of article, tapering strongly past apex, tapering gently to proximal end. G2 (Fig. 3E) distal article slightly shorter than basal article, slender, with tip truncated; basal article outer margin expanded.

**Female characters**. Female thoracic sternum (Fig. 4C), notably sternites 3, 4, relatively broad, surface setose. Sternites 1, 2 completely fused to form broadly triangular plate; separated from sternite 3 by concave suture; sternites 3, 4 fused, without trace of cleft demarcating suture, medially interrupted by median longitudinal line; sutures 4/5, 5/6, 6/7, 7/8 medially interrupted; median longitudinal groove visible at level of sternites 7, 8 only. Female sternopleonal cavity (Fig. 4C) shallow, reaching imaginary line joining median part of cheliped coxae; tubercle of sternopleonal locking mechanism (press-button) absent. Female pleon (Fig. 4B) longitudinally ovate in adults, covering most of thoracic sternum with all somites and telson free, somite 3–6 transversely rectangular, much wider than long, lateral margins convex. Vulva (Fig. 4C) rhomboid to oval shaped, large, recessed, opening directed mesial-ventrally, located mesial end of sternite 6, impinging on suture 5/6.

##### Remarks.

Yeo and Ng (1998), later followed by Yeo and Naruse (2008), mentioned that, based on personal communication with N. T. Đặng, the type specimens of this species are probably lost. Moreover, when establishing the substitute generic name, Đặng (2012) provided no information on whether any materials had been examined or was even available. As such, we concur that the type material of this species is most likely lost. Therefore, to stabilise the taxonomy of the species and the genus *Vietorientalia*, the present male topotype (46.1 × 35.4 mm; ZRC 2024.0291), collected from the type locality in Hoa Binh province (now part of Phú Thọ province), is hereby designated as the neotype of the species.

The differences between this species and allied species are addressed in the remarks on the genera *Vietorientalia* and *Dangmon* gen. nov.

##### Distribution.

Phú Thọ province, northern Vietnam.

#### Dangmon gen. nov.

Taxon classificationAnimaliaDecapodaPotamidae

A87C16A7-EFE7-58E5-8053-1BA8C62DA8BA

https://zoobank.org/F7AB6A74-5EE5-435A-9997-E3178CE5C66F

Figs 5, 6, 7, 8, 9

##### Type species.

*Orientalia rubra* Đặng & Trần, 1992, by present designation. Gender of genus neuter.

##### Diagnosis.

**Figure 5.**
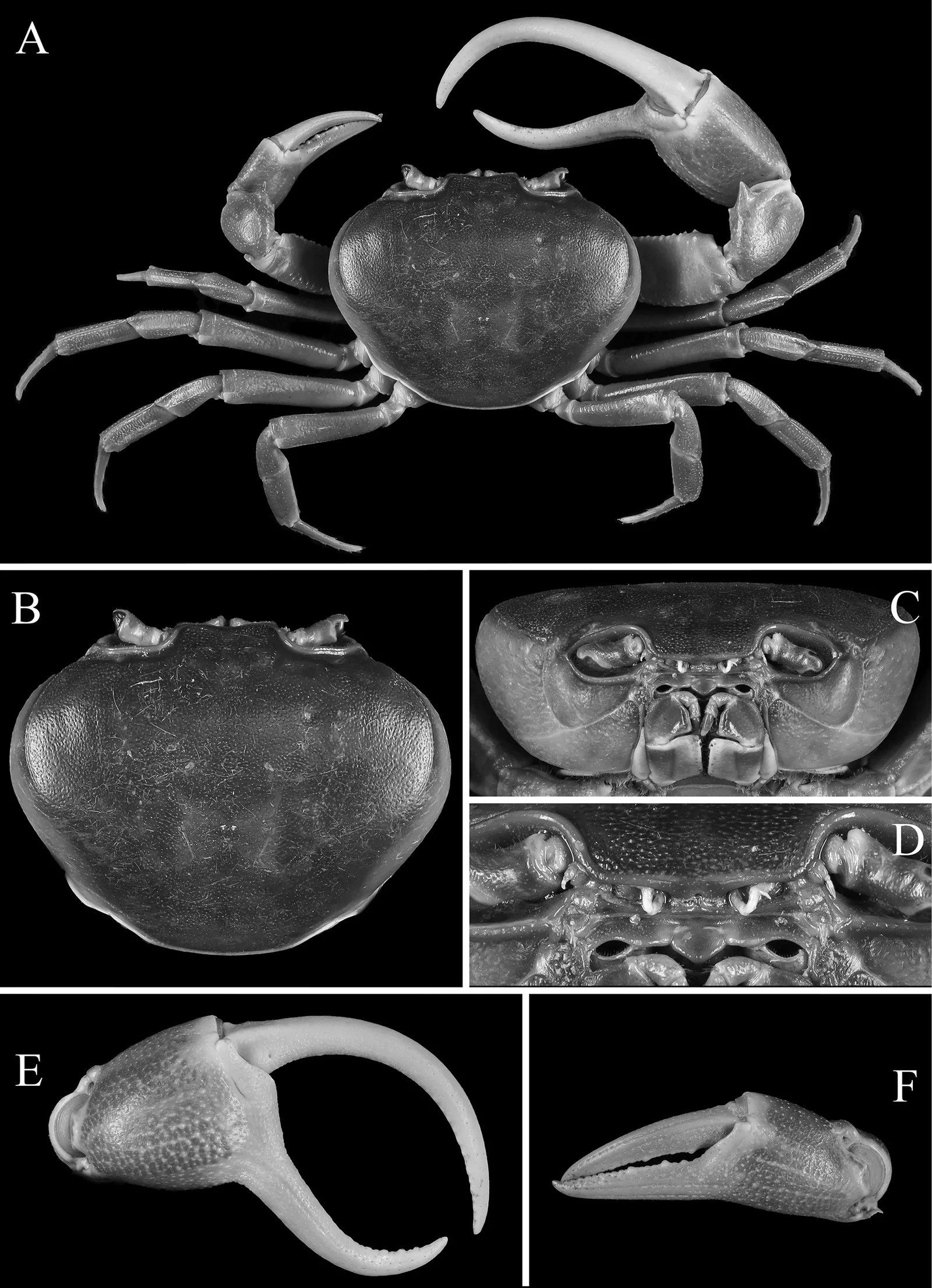
*Dangmon rubrum* (Đặng & Trần, 1992), gen. et comb. nov., male (52.8 × 40.3 mm) (ZRC 2022.0802), topotype, Thanh Hóa province, Vietnam. **A**. Overall dorsal habitus; **B**. Dorsal view of cephalothorax; **C**. Frontal view of cephalothorax; **D**. Antennae, antennules and epistome; **E**. Major chela (right); **F**. Minor chela (left).

**Figure 6.**
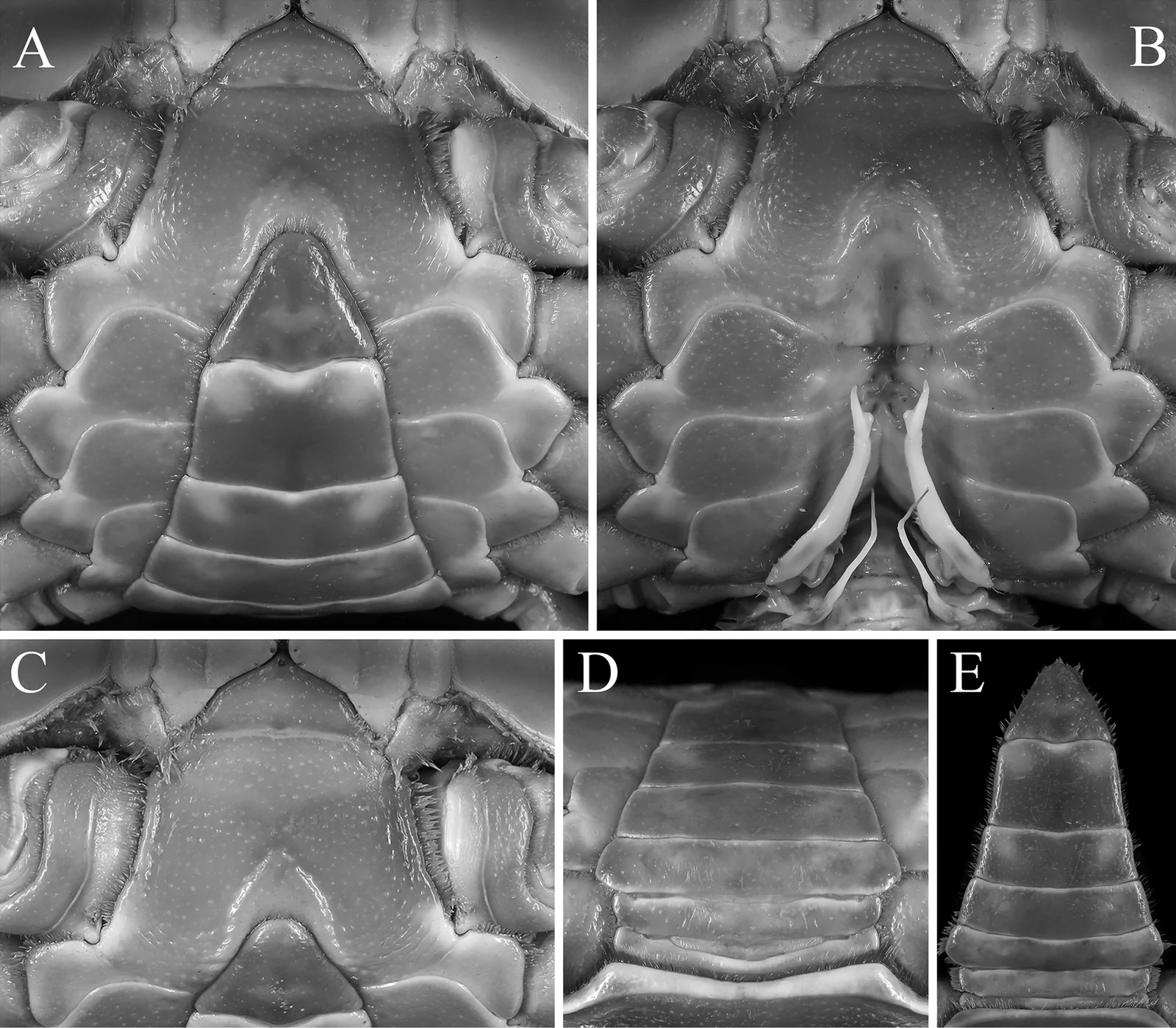
*Dangmon rubrum* (Đặng & Trần, 1992), gen. et comb. nov., male (52.8 × 40.3 mm) (ZRC 2022.0802), topotype, Thanh Hóa province, Vietnam. **A**. Thoracic sternites, pleon and telson; **B**. Sternopleonal cavity with G1 and G2 in-situ; **C**. Anterior thoracic sternites 1–4; **D**. Posterior thoracic sternum and pleon; **E**. Pleon.

**Figure 7.**
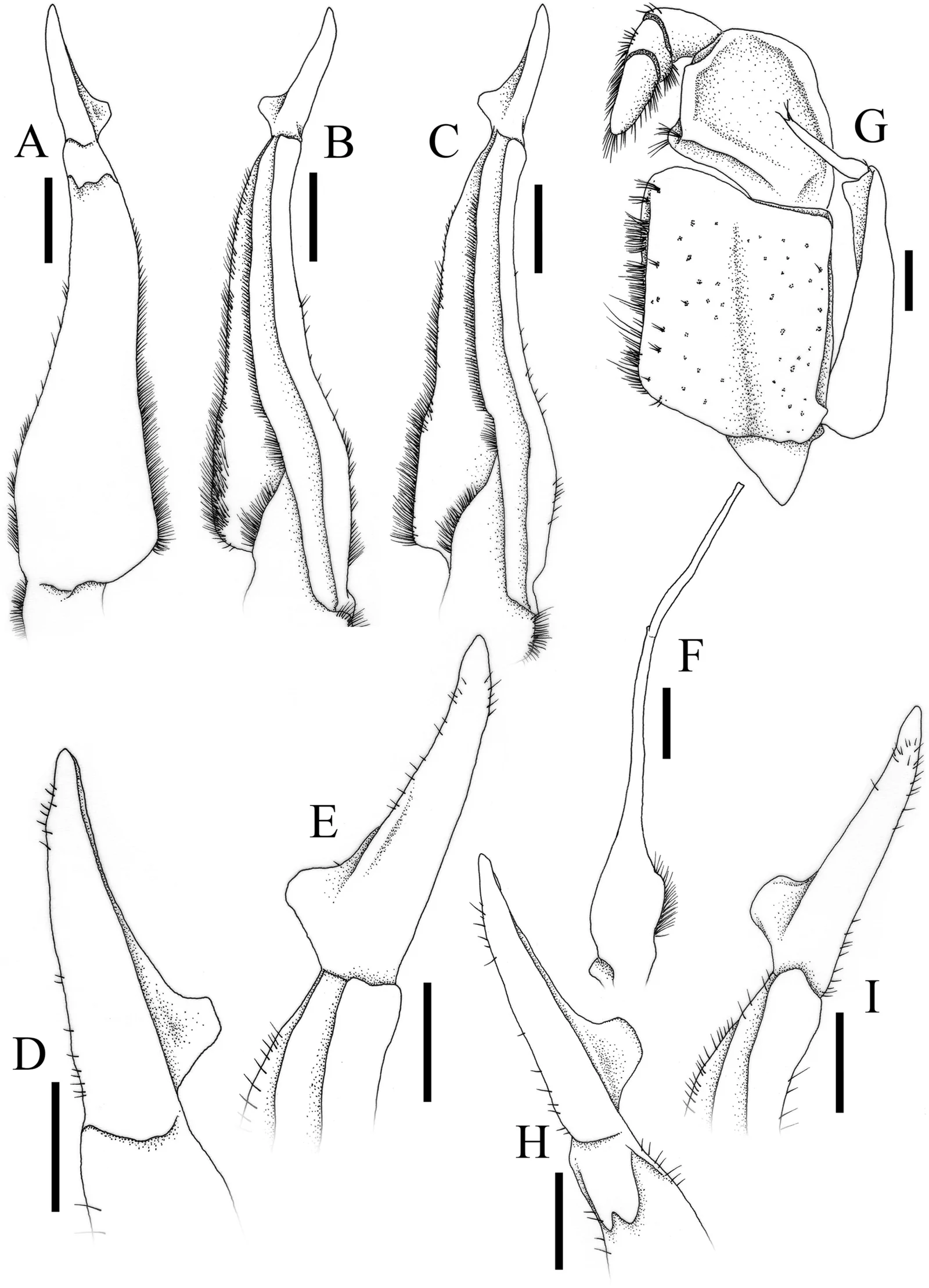
*Dangmon rubrum* (Đặng & Trần, 1992), gen. et comb. nov. **A–G**. Male (52.8 × 40.3 mm) (ZRC 2022.0802), topotype, Thanh Hóa province, Vietnam; **H, I**. Male (51.2 × 40.1 mm) (ZRC 2024.0292), obtained through aquarium trade from “northern Vietnam”; **A**. Left G1 dorsal view; **B**. Left G1 ventral view; **C**. Left G1 ventral view (obliquely orientated); **D, H**. Left G1 terminal article dorsal view; **E, I**. Left G1 terminal article ventral view; **F**. Left G2; **G**. Left third maxilliped. Scale bars: 2 mm (**A**–**C, F, G**); 1 mm (**D, E, H, I**).

**Figure 8.**
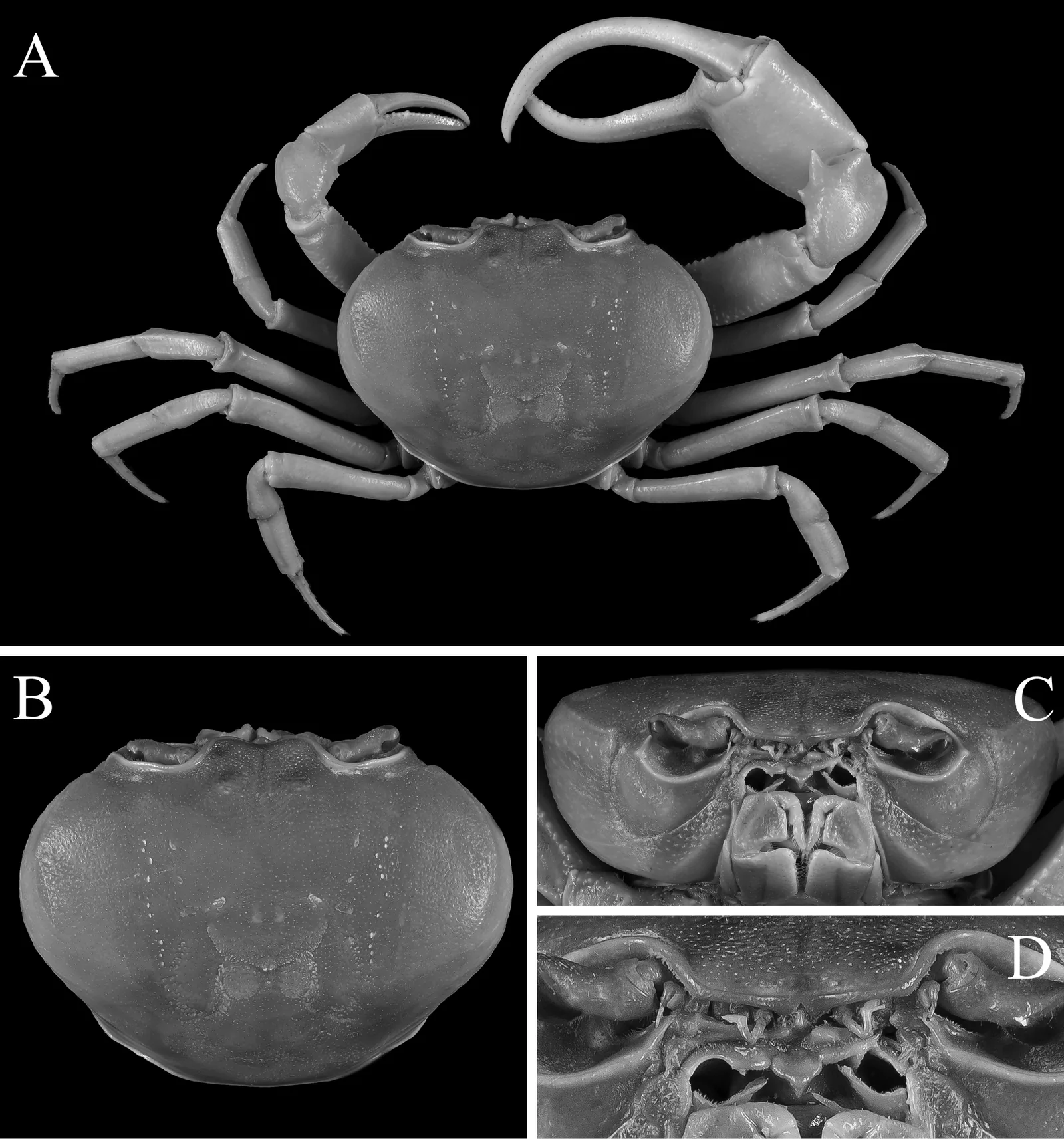
*Dangmon rubrum* (Đặng & Trần, 1992), gen. et comb. nov., male (51.2 × 40.1 mm) (ZRC 2024.0292), obtained through aquarium trade from “northern Vietnam”. **A**. Overall dorsal habitus; **B**. Dorsal view of cephalothorax; **C**. Frontal view of cephalothorax; **D**. Antennae, antennules and epistome.

**Figure 9.**
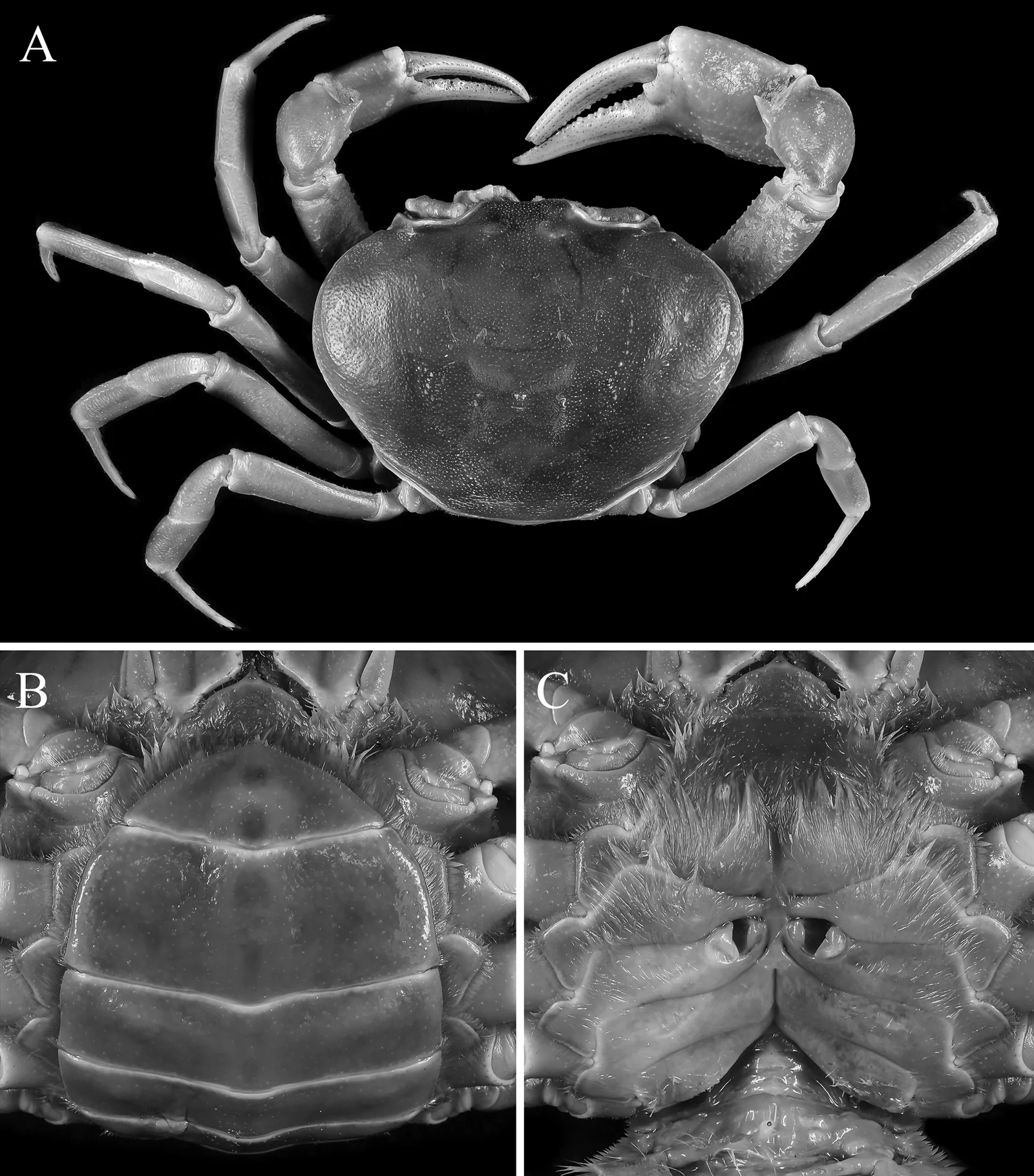
*Dangmon rubrum* (Đặng & Trần, 1992), gen. et comb. nov., female (52.8 × 40.5 mm) (ZRC 2022.0802), topotype, Thanh Hóa province, Vietnam. **A**. Overall dorsal habitus; **B**. Pleon and telson; **C**. Sternopleonal cavity and vulvae.

Carapace (Figs 5A–C, 8A–C, 9A) distinctly broader than long, high; dorsal surface strongly convex longitudinally, convex transversely, smooth, grooves indistinct, regions not well defined; epigastric cristae almost indiscernible, slightly anterior and confluent with postorbital cristae; postorbital cristae very weak, rounded; external orbital tooth triangular, low but discernible, separated from epibranchial tooth by weak, broad cleft; epibranchial tooth appearing as rounded angle; anterolateral margin distinctly convex, completely smooth. Third maxilliped (Fig. 7G) with short flagellum, just exceeding half merus width. Chelipeds in adult males (Figs 5A, 5E, 5F, 8A) unequal, fingers of major chela gaping strongly; chelipeds in adult females (Fig. 9A) more symmetrical in size with fingers less strongly gaping; ambulatory legs (Figs 5A, 8A, 9A) elongate, with long, slender dactyli. Thoracic sternites 3 and 4 (Fig. 6A, C) with very shallow groove demarcating suture; male sternopleonal cavity exceeds imaginary line joining medial parts of cheliped coxae. G1 (Fig. 7A–E, H, I) long, slender, gently sinuous; terminal article slender, subconical, distally gently curving inwards, with relatively low dorsal flap in proximal part.

##### Etymology.

The new genus is named after the late Professor Đặng Ngọc Thanh (Vietnam National Centre for Natural Science and Technology) in honour of his long and substantial contributions to the field of carcinology in Vietnam. The gender of the genus is neuter.

##### Remarks.

The new genus, *Dangmon* gen. nov., is currently represented only by *D. rubrum* (Đặng & Trần, 1992), comb. nov., which was previously placed in the genus *Vietorientalia* Đặng, 2012, which is now also monotypic, represented solely by *V. glabra* (Đặng, 1967). *Dangmon* gen. nov. can be differentiated from *Vietorientalia* by the following differences in external morphology and G1 morphology: carapace relatively lower, less inflated (Figs 5A, 5C, 8A, 8C) (vs carapace higher, appearing more inflated, Fig. 1A, C); carapace convex transversely, branchial regions not prominently inflated (Figs 5C, 8C) (vs carapace being less convex transversely, branchial regions inflated, Fig. 1C); anterior thoracic sternum narrower transversely, more elongate longitudinally, with smooth surface (Fig. 6C) (vs anterior thoracic sternum wider transversely, wider than long, with densely pitted surface, Fig. 2C); major cheliped fingers in adult males elongate, slender and distinctly gaping (Figs 5E, 8A) (vs major cheliped fingers in adult males relatively short, stout and not gaping, Fig. 1E); ambulatory legs less elongate, with relatively stouter dactyli (Figs 5A, 8A, 9A) (vs ambulatory legs more elongate, with distinctly elongate and slenderer dactyli, Figs 1A, 4A); and G1 terminal article distally curving gently inwards, with a relatively low dorsal flap on the proximal part (Fig. 7D, E, H, I) (vs G1 terminal article curving outwards, with a prominent and higher dorsal flap on the proximal part, Fig. 4C, D).

**Figure 10.**
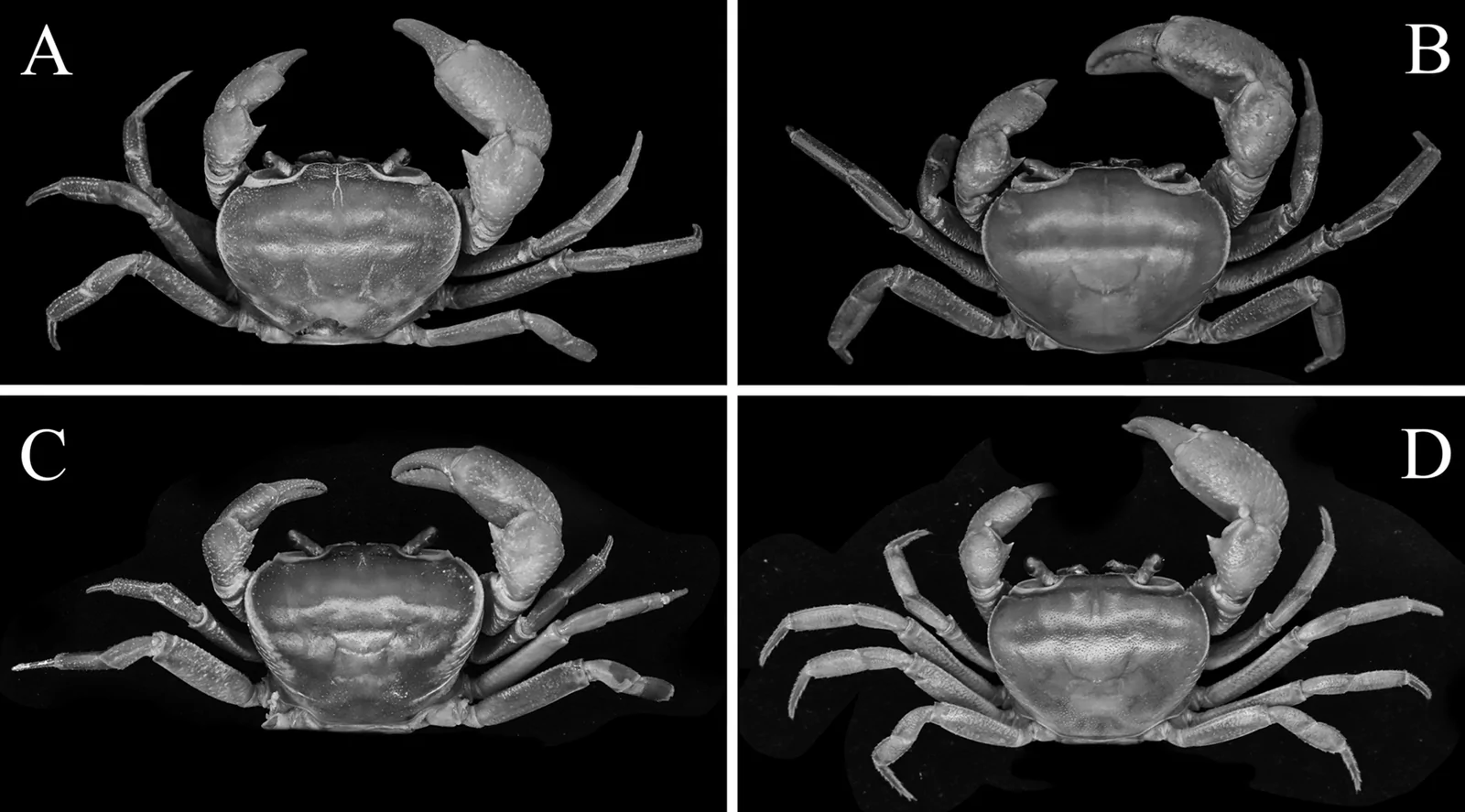
Dorsal views of *Hainanpotamon* species from Hainan Island, China. **A**. *H. orientale* (Parisi, 1916), male (37.2 × 25.1 mm) (NNU5484-01); **B**. *H. fuchengense* Dai, 1995, male (30.1 × 20.8 mm) (NNU5481-01); **C**. *H. helense* Dai, 1995, male (36.3 × 28.2 mm) (NNU5491-01); **D**. *H. daiae* Yeo & Naruse, 2008, male (30.4 × 24.8 mm) (NNU5443-01).

**Figure 11.**
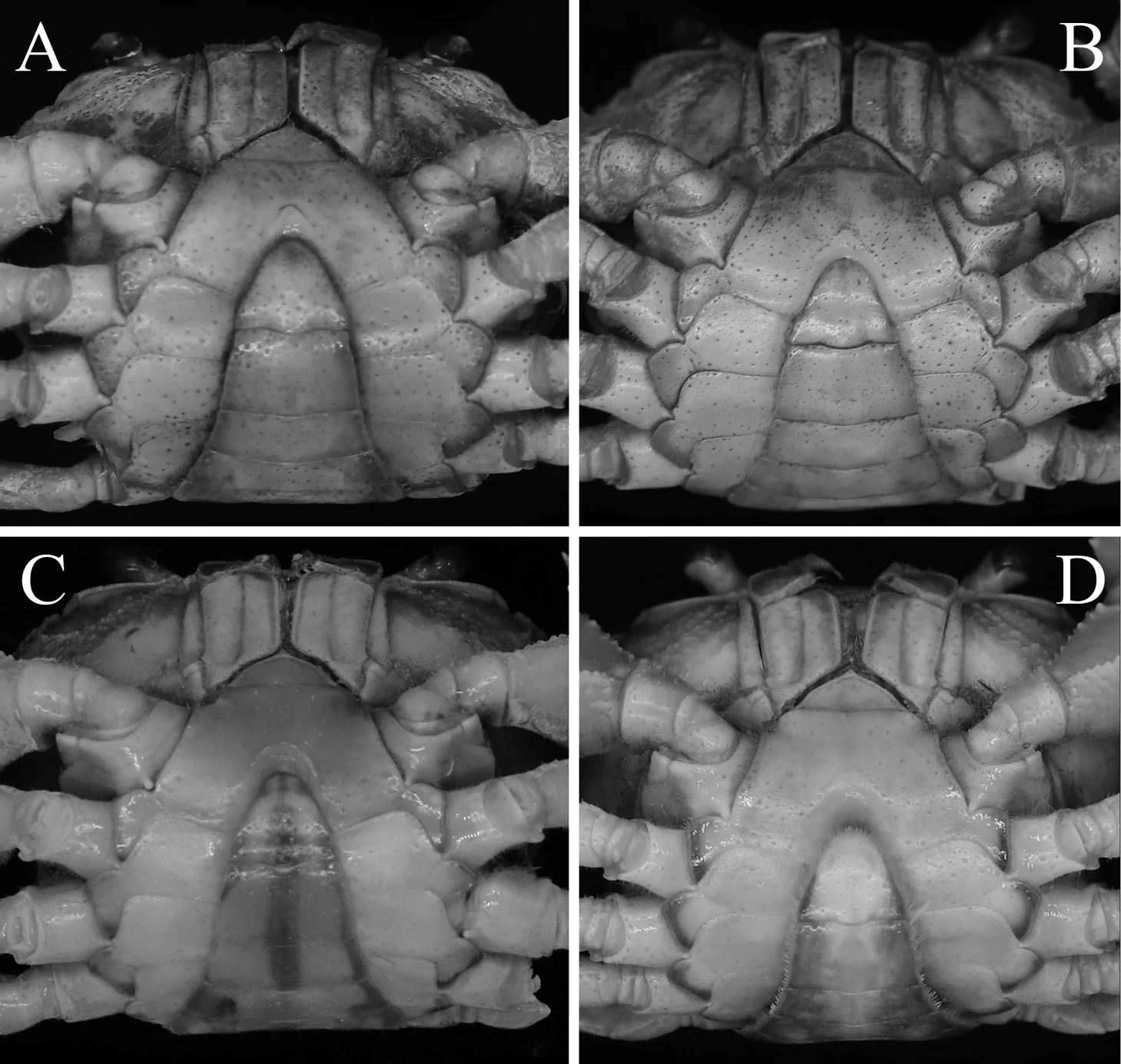
Ventral views showing sternum, pleon and telson of *Hainanpotamon* species from Hainan Island, China. **A**. *H. orientale* (Parisi, 1916), male (37.2 × 25.1 mm) (NNU5484-01); **B**. *H. fuchengense* Dai, 1995, male (30.1 × 20.8 mm) (NNU5481-01); **C**. *H. helense* Dai, 1995, male (36.3 × 28.2 mm) (NNU5491-01); **D**. *H. daiae* Yeo & Naruse, 2008, male (30.4 × 24.8 mm) (NNU5443-01).

Both *Dangmon* gen. nov. and *Hainanpotamon* sensu stricto (*H. orientale*, *H. fuchengense*, *H. helense*, and *H. daiae*) feature an inflated, globose carapace and slender, elongate G1 that possesses a low dorsal flap only in the proximal portion of the slender conical terminal article (Figs 5A, 5C, 7A–E, 7H, 7I, 8A, 8C, 10, 13A, 13B, 13D, 13E, 13G, 13H, 13J, 13K; cf. Yeo and Naruse 2008: figs 3, 5C–F). The sole species of the genus, *Dangmon rubrum* comb. nov., however, displays clear differences in its external morphology that warrant the establishment of the new genus and distinguishes it from *Hainanpotamon* sensu stricto by the following morphological differences: anterolateral margin completely smooth (Figs 5A, 8A, 9A; cf. Yeo and Naruse 2008: fig. 6A, B) (vs anterolateral margin cristate, appearing serrated, Fig. 10; cf. Yeo and Naruse 2008: figs 1A, 4A); epibranchial tooth almost indiscernible, appearing as a smooth angle or completely indiscernible (Figs 5A, 8A, 9A; cf. Yeo and Naruse 2008: fig. 6A) (vs epibranchial tooth well defined, or as a distinct angle, Fig. 10; cf. Yeo and Naruse 2008: figs 1A, 4A); pterygostomial, subhepatic, sub-branchial regions very weakly rugose and suborbital region smooth (Figs 5C, 8C; cf. Yeo and Naruse 2008: fig. 6B) (vs pterygostomial, subhepatic, sub-branchial and suborbital regions strongly granulose and rugose, Fig. 11; cf. Yeo and Naruse 2008: figs 1B, 4B); third maxilliped flagellum not exceeding width of merus (Fig. 7G) (vs third maxilliped flagellum well produced, exceeding width of merus, cf. Yeo and Naruse 2008: figs 2A, 5A); and cheliped surfaces smooth, fingers very slender and long in male major chela (Figs 5E, 5F, 8A; cf. Yeo and Naruse 2008: fig. 6A, B) (vs cheliped surfaces strongly granulose, fingers stouter and shorter, Fig. 10; cf. Yeo and Naruse 2008: figs 1, 2C, 4).

Another potamid genus having a globose appearance found in northern Vietnam is *Lacunipotamon* Dai, Song, He, Cao, Xu & Zhong, 1975 (see Ng et al. 2023). *Lacunipotamon*, however, is clearly differentiated from *Dangmon* gen. nov. by its stouter and shorter G1, with a terminal article tip that is recurved or dilated (cf. Ng et al. 2023: figs 2, 5, 31, 47), whereas in *Dangmon* gen. nov. it is slenderer and more elongate, with a subconical terminal article (Fig. 7A–E, H, I).

##### Distribution.

Thanh Hóa province, northern Vietnam.

#### Dangmon rubrum

Taxon classificationAnimaliaDecapodaPotamidae

(Đặng & Trần, 1992) comb. nov.

FF2B7D6D-8EAA-5A40-93FA-A21ABB9213FF

Figs 5, 6, 7, 8, 9

Orientalia rubra Đặng & Trần, 1992: 17, fig. 1.
Hainanpotamon rubrum —Yeo and Ng 1998: 358.—Yeo and Naruse 2008: 1150.—Ng et al. 2008: 162.—Do 2014: 111.
Hainanpotamon rubra —Yeo and Ng 1999: 641.
Vietorientalia rubrum —Đặng 2012: 307.—Đặng and Hồ 2012: 191.

##### Material examined.

***Non type specimens***: Vietnam • 1 male (52.8 × 40.3 mm), 1 female (52.8 × 40.5 mm); stream bank of bamboo plantation hill, Phùng Giáo commune, Ngọc Lặc district, Thanh Hóa province, northern Vietnam; 14 Aug. 2022; Ngo Van Tri leg.; ZRC 2022.0802 • 5 males (38.0 × 29.5 mm, 46.6 × 36.6 mm, 47.1 × 37.6 mm, 48.7 × 37.2 mm, 51.2 × 40.1 mm), 4 females (42.2 × 33.0 mm, 47.0 × 37.5 mm, 48.1 × 37.1 mm, 54.6 × 42.4 mm); ornamental trade material purchased from aquarium shops in Singapore, imported from Vietnam; 20 Apr. 2014; purchased by K.K.P. Lim & H.H. Tan; ZRC 2024.0292.

##### Description.

Carapace (Figs 5A, 5B, 8A, 9A) transversely ovate, distinctly wider than long (CW/CL ratio: 1.25–1.31), high; dorsal surface convex transversely, strongly convex longitudinally, completely smooth but finely punctate. Frontal regions finely punctate, orbital regions smooth; suborbital region smooth; pterygostomial, subhepatic, sub-branchial regions gently rugose. Epigastric cristae smooth, almost indiscernible, appearing as two low humps; postorbital cristae smooth, rounded, confluent with epibranchial tooth and anterolateral margins. H-shaped median groove, cervical grooves indiscernible. Frontal margin straight, appears entire in dorsal view, strongly deflexed downwards, gently convex in frontal view. External orbital tooth very weakly produced, broadly triangular, outer margin straight, slightly more than length of inner margin, demarcated from rest of anterolateral margin by broad, shallow cleft. Epibranchial tooth indistinct, appearing as rounded angle. Anterolateral margins strongly convex, with trace of cristae, otherwise smooth. Posterolateral margins strongly converging towards posterior carapace margin.

Orbits (Figs 5C, 8C) large, ovate; eye partially filling orbital space; ocular peduncle relatively short; cornea large, ovate, pigmented. Supraorbital margin almost straight, cristate, smooth. Suborbital margin concave, cristate, with low tubercles. Antennae very short, barely produced; antennules short, folding transversely in subrectangular fossa. Posterior margin of epistome with distinct, broadly triangular median tooth, inner margins straight, outer margins concave.

Third maxillipeds (Figs 5C, 7F, 8C) covering most of buccal cavity when closed; ischium subrectangular, with distinct median groove, surface very sparsely pitted, otherwise smooth; merus subquadrate, nearly as wide as long, surface smooth, margins weakly cristate; exopod slender, reaching to about one-third length of merus, inner subdistal portion produced, with flagellum reaching slightly more than half of merus width, but not exceeding width of merus.

Chelipeds (Figs 5A, 5E, 5F, 8A, 9A) strongly unequal, especially in males. Basis-ischium anterior margin lined with small, rounded granules, otherwise smooth; merus with margins lined with low rounded granules, appearing serrated; carpus dorsal surface smooth with few granules, inner distal angle with large sharp tooth, basal tooth produced. Chelae with outer surface smooth, finely punctate. Major chela appearing more strongly inflated, proportionally higher, minor chela appearing less strongly inflated, proportionally shorter. Major chela with fingers long, slender, longer than palm, forming prominent gape when fingers closed, especially in males; small gape in minor chela; cutting edges of both fingers with few sharp teeth and denticles.

Ambulatory legs (Figs 5A, 8A, 9A) elongate; second pair longest, last pair shortest. Merus long, slender, outer surface smooth, dorsal margin entire, sub-cristate, without subdistal spine or tooth; carpus smooth, dorsal margin smooth, dorsal surface with very weak sub-median crista; propodus with dorsal margin sub-cristate, dorsal surface with very weak sub-median groove; dactylus elongate, distally gently curving, trapezoidal in cross section, slightly longer than propodus, margins with sharp spines.

Thoracic sternum (Fig. 6A, C), notably sternites 3, 4, relatively elongate, surface smooth. Sternites 1, 2 completely fused to form broadly triangular plate, separated from sternite 3 by distinct, straight suture; sternites 3, 4 fused, with very shallow but complete demarcating suture. Sutures 4/5, 5/6, 6/7, 7/8 medially interrupted; median longitudinal groove on sternites 7, 8. Male sternopleonal cavity (Fig. 6B) deep, exceeding imaginary line joining medial part of cheliped coxae. Tubercle of sternopleonal locking mechanism (press-button) located posteriorly on sternite 5, closer to suture 5/6.

Male pleon (Fig. 6D, E) triangular, all somites, telson free; telson triangular, lateral margins straight for most part; somite 6 trapezoidal, slightly wider than long, lateral margins straight; somites 3–5 trapezoidal, gradually decreasing in width, increasing in length; somites 1, 2 subrectangular, very wide, reaching to bases of coxae of fourth ambulatory legs, thoracic sternite 8 not visible when pleon closed.

G1 (Fig. 7A–E, H, I) gently sinuous, slender; subterminal article broader proximally, tapering distally, without any subrectangular cleft on upper part of outer margin; terminal article very gently bent outwards, clearly separated from subterminal article, relatively slender, about one-third times length of subterminal article, conical, distally gently curved inwards, groove for G2 sub-marginal, with low trapezoidal, subrectangular dorsal flap at proximal region. G2 (Fig. 7F) distal article distinctly longer than half of basal article, slender, tapering, with tip truncated; basal article outer margin expanded.

**Female characters**. Female thoracic sternum (Fig. 9C), notably sternites 3, 4, relatively broad, surface densely setose. Sternites 1, 2 completely fused to form broadly triangular plate, separated from sternite 3 by straight, distinct suture; sternites 3, 4 fused, without any groove demarcating suture, medially interrupted by median longitudinal line at posterior half; sutures 4/5, 5/6, 6/7, 7/8 medially interrupted; median longitudinal groove visible at level of sternites 7, 8 only. Female sternopleonal cavity (Fig. 9C) shallow, reaching imaginary line joining medial part of cheliped coxae; tubercle of sternopleonal locking mechanism (press-button) absent. Female pleon (Fig. 9B) longitudinally ovate in adults, covering most of thoracic sternum, with all somites and telson free; somite 3–6 transversely rectangular, much wider than long, lateral margins convex. Vulva (Fig. 9C) transversely ovate, large, recessed, opening directed mesial-ventrally, located mesial end of sternite 6, impinging on suture 5/6.

##### Remarks.

The type series of *Dangmon rubrum* comb. nov. was not available for examination during the course of this study. Nevertheless, the material examined herein comprises one male and one female collected from the vicinity of the type locality and additional material purchased from aquarium shops in Singapore. All material examined agrees well with the original and subsequent descriptions and figures provided by Đặng and Trần (1992) and Yeo and Naruse (2008). The exact collection locality of the aquarium specimens is not known. However, the suppliers of the ornamental trade material in Singapore informed us that the specimens were collected from “northern Vietnam”. In terms of morphology, the topotypes show some intraspecific differences from the material obtained through the aquarium trade. Some of the ambulatory legs of the topotype male appear to be much shorter than those of the topotype female and aquarium-trade material (Figs 5A, 8A, 9A). This discrepancy could be due to a teratological condition resulting from previous limb loss and/or moulting. In addition, the G1 terminal article and the shape of the dorsal flap of the specimens show some variation. The G1 terminal article in the topotype male is slightly broader and the dorsal flap being more trapezoidal in shape (Fig. 7D, E), compared with the slightly slenderer G1 terminal article and a more rounded, subrectangular dorsal flap observed in the aquarium-trade specimen (Fig. 7H, I). Otherwise, the specimens show very little differences in other aspects of their external morphology and live colouration. As such, the specimens are all regarded herein as *Dangmon rubrum* (Đặng & Trần, 1992), comb. nov.

##### Distribution.

Thanh Hóa province, northern Vietnam.

#### Hainanpotamon

Taxon classificationAnimaliaDecapodaPotamidae

Dai, 1995

556CF128-CFD4-56DB-8C33-0E9B511E3191

Figs 10, 11, 12, 13

Hainanpotamon Dai, 1995: 391—Dai 1999: 145.—Yeo and Ng 1998: 357.—Yeo and Ng 2005: 12.—Yeo and Naruse 2008: 1143.—Ng et al. 2008: 162.—Ng et al. 2023: 2, 4.

##### Type species.

Potamon (Potamon) orientale Parisi, 1916, by original designation. Gender of genus neuter.

##### Material examined.

***Hainanpotamon orientale* Parisi, 1916. *Non-type specimens***: China • 2 males (35.3 × 23.2 mm, 37.2 × 25.1 mm); 1 female (34.8 × 22.7 mm); Nankun Township, Tunchang County, Hainan Province, China; 19.30°N, 109.93°E; 148 m asl; 19 Dec. 2012; Cha He, Qiang Zhao, and Hongying Sun leg.; NNU5484-01–03 • 3 males (31.7 × 22.8 mm, 34.5 × 24.2 mm, 35.2 × 25.7 mm); Baisha County, Hainan Province, China; 18.62°N, 109.67°E; 213 m asl; 4 Jan. 2013; Cha He, Qiang Zhao, and Hongying Sun leg.; NNU5433-01–03.

**Figure 12.**
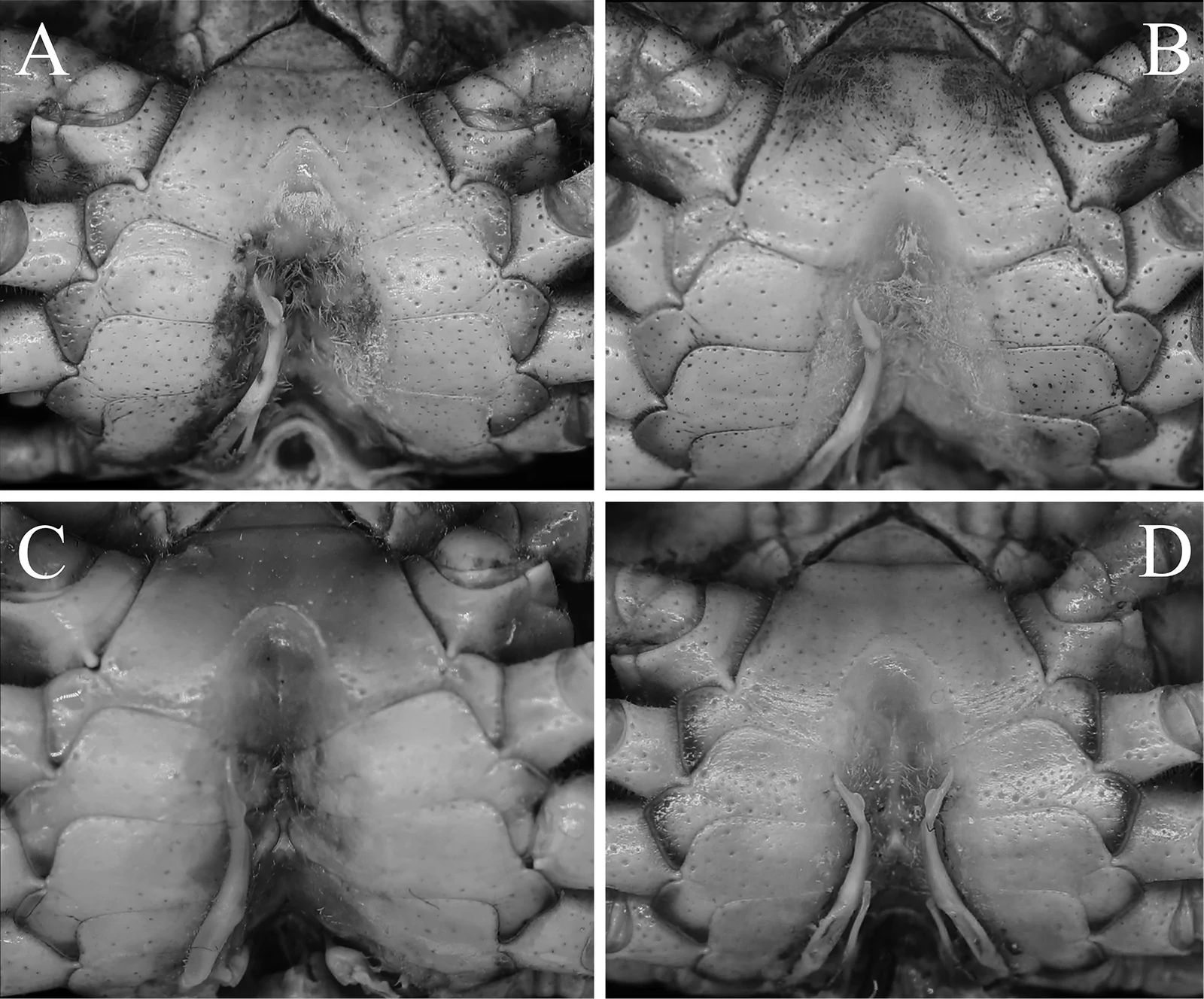
Sternopleonal cavity with G1 and G2 in-situ in *Hainanpotamon* species from Hainan Island, China. **A**. *H. orientale* (Parisi, 1916), male (37.2 × 25.1 mm) (NNU5484-01); **B**. *H. fuchengense* Dai, 1995, male (30.1 × 20.8 mm) (NNU5481-01); **C**. *H. helense* Dai, 1995, male (36.3 × 28.2 mm) (NNU5491-01); **D**. *H. daiae* Yeo & Naruse, 2008, male (30.4 × 24.8 mm) (NNU5443-01).

**Figure 13.**
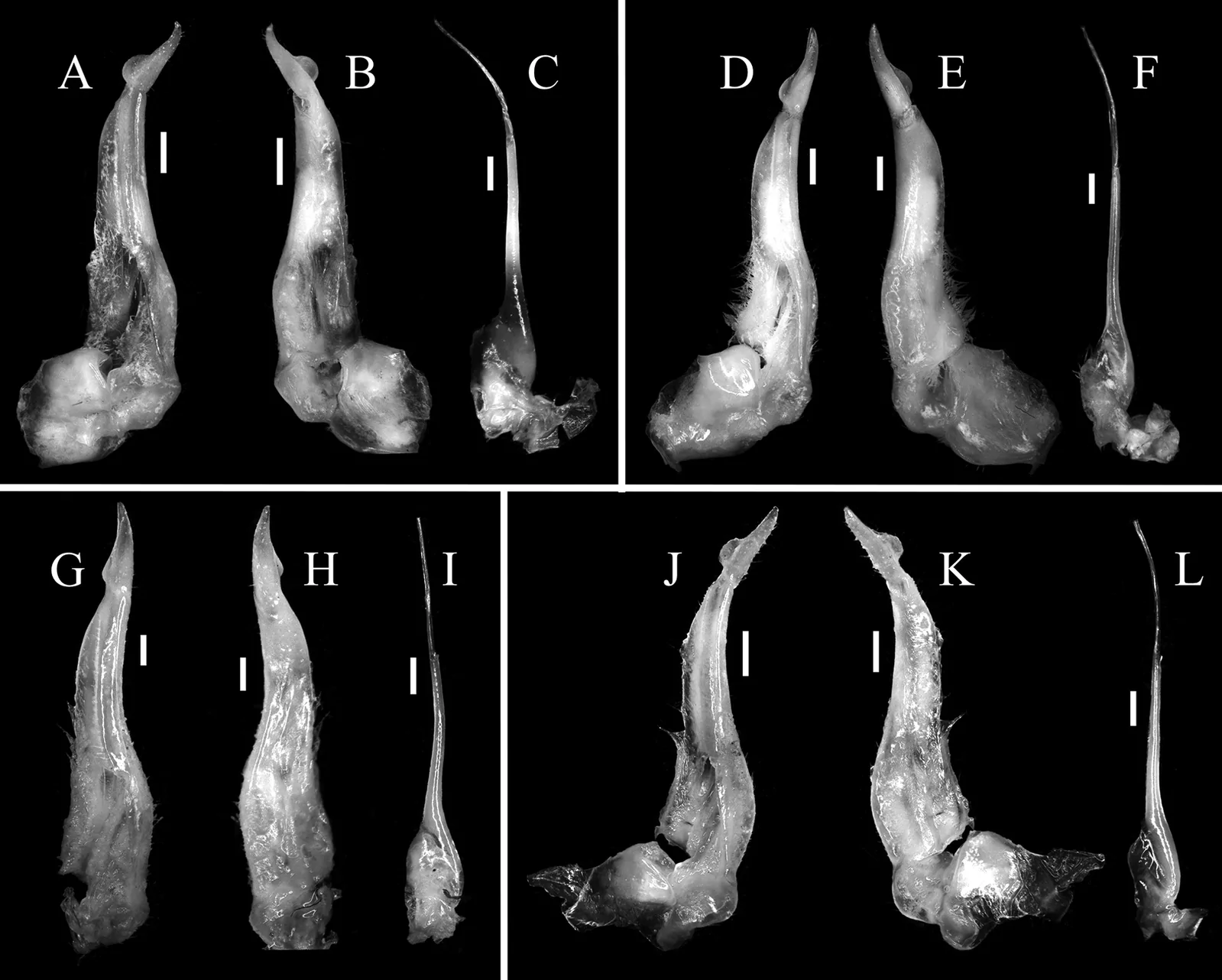
G1 and G2 of *Hainanpotamon* species from Hainan Island, China. **A–C**. *H. orientale* (Parisi, 1916), male (37.2 × 25.1 mm) (NNU5484-01); **D–F**. *H. fuchengense* Dai, 1995, male (30.1 × 20.8 mm) (NNU5481-01); **G–I**. *H. helense* Dai, 1995, male (36.3 × 28.2 mm) (NNU5491-01); **J–L**. *H. daiae* Yeo & Naruse, 2008, male (30.4 × 24.8 mm) (NNU5443-01); **A, D, G, J**. Left G1 dorsal view; **B, E, H, K**. Left G1 ventral view; **C, F, I, L**. Left G2. Scale bar: 1 mm.

***Hainanpotamon fuchengense* Dai, 1995. *Non-type specimens***: China • 2 males (25.1 × 18.9 mm, 30.1 × 20.8 mm); 2 females (31.1 × 25.2 mm, 32.5 × 25.0 mm); Mutang Village, Huishan Town, Qionghai City, Hainan Province, China; 19.06°N, 110.28°E; 46 m asl; 21 Dec. 2012; Cha He, Qiang Zhao, and Hongying Sun leg.; NNU5481-01–04.

***Hainanpotamon helense* Dai, 1995. *Non-type specimens***: China • 3 males (32.2 × 25.1 mm, 36.3 × 28.2 mm, 33.1 × 23.8 mm); 1 female (42.6 × 35.8 mm); Longge Village, Qionghai, Hainan Province, China; 19.29°N, 110.45°E; 18 m asl; 23 Dec. 2008; Qiang Zhao, Wei Gao, and Hongying Sun leg.; NNU5491-01–04.

***Hainanpotamon daiae* Yeo & Naruse, 2008. *Non-type specimens***: China • 4 males (30.7 × 24.3 mm, 30.4 × 24.8 mm, 28.7 × 22.6 mm, 24.7 × 19.4 mm); 2 females (39.7 × 31.4 mm, 35.1 × 28.3); Maoding Village, Maoding Township, Baoting County, Hainan Province, China; 18.62°N, 109.67°E; 71 m asl; 23 Dec. 2008; Qiang Zhao, Wei Gao, and Hongying Sun leg.; NNU5443-01–06.

##### Remarks.

Yeo and Naruse (2008) recognised nine species of *Hainanpotamon*, with a broad distribution, encompassing Hainan Island (China), northern Laos, and Vietnam. *Hainanpotamon globosum* was described from Okinawa, Japan, but Yeo and Naruse (2008: 1150) argued that the locality data for the type series of *H. globosum* may have resulted from mislabelling. Based on our current morphological and molecular assessment, *Hainanpotamon* sensu Yeo and Naruse (2008) is not monophyletic. Thus, following the principle of typification, the genus *Hainanpotamon* sensu stricto is characterised by the diagnostic characters of the type species, *H. orientale* (Parisi, 1916), and other closely related species distributed on Hainan Island, viz., *H. fuchengense*, *H. helense*, and *H. daiae*. The recognition of *Vietorientalia* Đặng, 2012, for its type species, *V. glabra* (Đặng, 1967), which was previously placed in *Hainanpotamon* by Yeo and Naruse (2008), is well supported by morphological and molecular analyses. This study also found it necessary to establish the monotypic genus *Dangmon* gen. nov. for *Vietorientalia rubra* (Đặng & Trần, 1992), which was also previously placed in *Hainanpotamon* sensu Yeo and Naruse (2008). The differences among *Vietorientalia*, *Dangmon* gen. nov., and *Hainanpotamon* sensu stricto are discussed in detail in the remarks on the genera *Vietorientalia* and *Dangmon* gen. nov.

The remaining seven species of *Hainanpotamon* sensu Yeo and Naruse (2008) now have a disjunct distribution, with four species (*H. orientale*, *H. fuchengense*, *H. helense*, and *H. daiae*) on Hainan Island, *H. globosum* purportedly from Okinawa Island, *H. auriculatum* from central Vietnam, and *H. directum* from northern Laos. Preliminary unpublished morphological and molecular analyses suggest that the Indochinese species do not form a monophyletic group with the remaining *Hainanpotamon* species (sensu stricto). As such, the morphological treatments of *Vietorientalia* and *Dangmon* gen. nov. are undertaken only in comparison with *Hainanpotamon* species from Hainan Island in the present study. The generic placement of *Hainanpotamon globosum* (Parisi, 1916) (after Yeo and Naruse 2008) is also contentious, as Ng et al. (2008), in their checklist of all extant brachyuran crab species of the world, listed the species as *Nanhaipotamon globosum* (Parisi, 1916). The formal treatment and placement of *Nanhaipotamon globosum* with the remaining species of *Hainanpotamon* sensu lato will be addressed in a separate revision.

## Discussion

**Figure 14.**
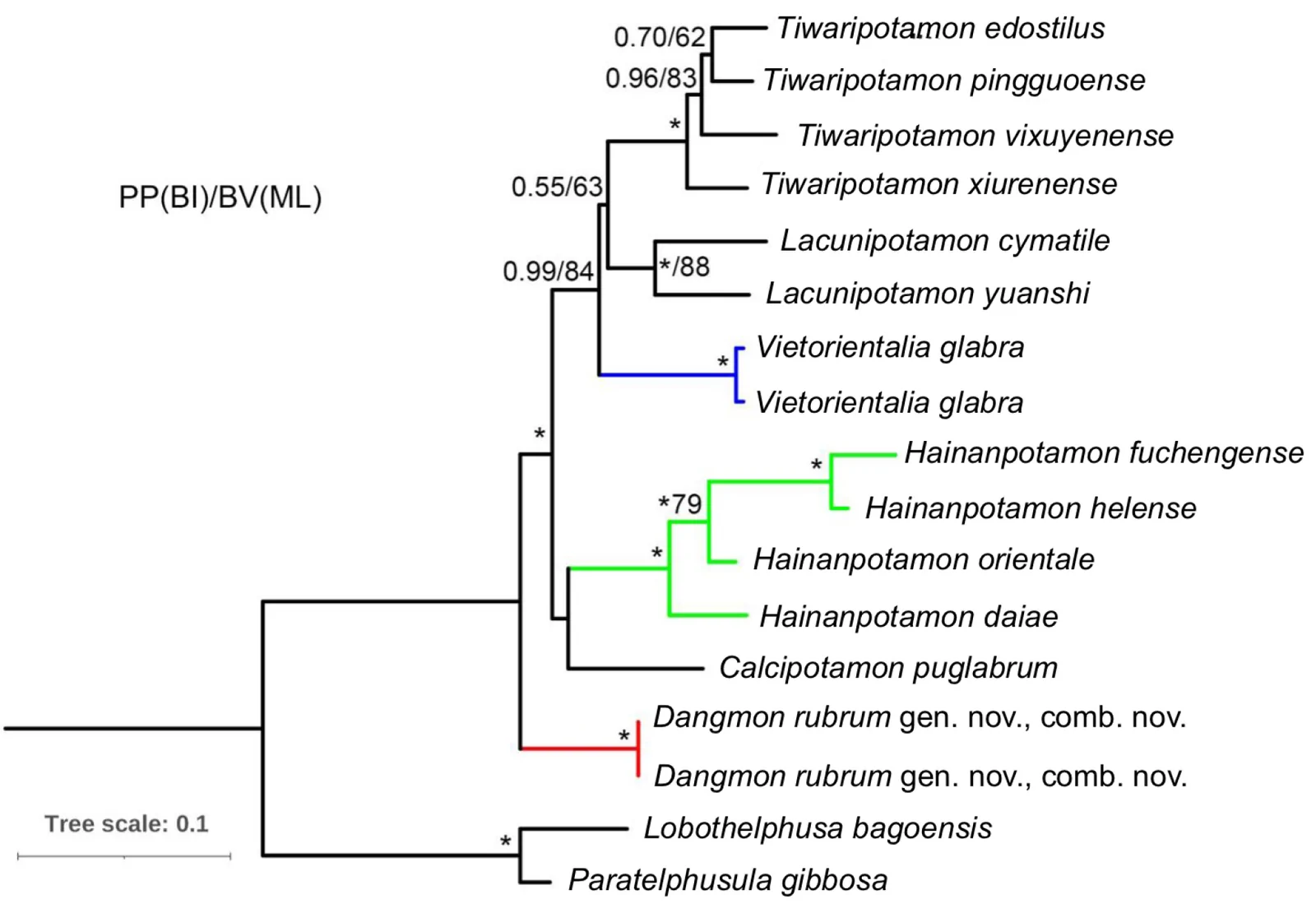
The phylogenetic topology for the freshwater crabs treated in this study and other closely related species, based on four DNA sequences (16S, COI, 28S, H3). Values (BS/PP) at nodes represent bootstrap values (BS) and posterior probabilities (PP). Coloured branches indicate the different genera treated in this study. Asterisks indicate BS = 100 or PP = 1.00.

A total of 15 species representing seven genera and one new genus, including *Hainanpotamon* sensu stricto (represented in this study by Hainan Island endemics, viz., *H. orientale*, *H. fuchengense*, *H. helense*, and *H. daiae*) and other geographically related species, were included in the phylogenetic analysis. Sixty-eight sequences were used, of which 48 were newly obtained for this study (Suppl. material 2). The resultant matrix consists of 2,200 bp, of which 16S comprises 468 bp, COI 599 bp, 28S 895 bp, and H3 238 bp. ML and BI analyses resulted in congruent tree topologies with some minor differences in the terminal lineages. The inferred maximum likelihood topology is shown in Fig. 14, with the statistics from different analyses for the major nodes [PP/BV: posterior probabilities (BI) and bootstrap values (ML)]. Both the ML and BI trees generally received strong nodal support, with strong support also observed at deeper nodes (only three nodes had lower support values, PP and/or BV ≤ 75%).

All genera analysed are monophyletic and are supported by strong probability values (Fig. 14). *Hainanpotamon* sensu stricto, represented by the green lineage, consists of four species (*H. daiae*, *H. fuchengense*, *H. orientale*, and *H. helense*), which are endemic to Hainan Island in China. The genus is sister to *Calcipotamon puglabrum* Huang, Huang & Shen, 2020 [Hainan Island, China]. *Vietorientalia glabra* is recognised as a well-supported monophyletic lineage (blue) and forms a sister lineage to the *Lacunipotamon* and *Tiwaripotamon* species. *Dangmon rubrum* gen. et comb. nov., forms the basal lineage (red) and is genetically distinct from other closely related species. The combined phylogenetic evidence and morphological traits thus support the present taxonomic arrangement of these taxa.

## Supplementary Material

XML Treatment for Vietorientalia

XML Treatment for Vietorientalia glabra

XML Treatment for Dangmon

XML Treatment for Dangmon rubrum

XML Treatment for Hainanpotamon

## Additional information

### Artificial Intelligence (AI) use

The authors accept full responsibility for the content of the manuscript, including the disclosure of any use of AI.

No AI tools were used in the preparation of this manuscript.

### Author ORCIDs

Z.W. Tan https://orcid.org/0000-0002-9878-6958

V.T. Ngo https://orcid.org/0000-0002-0333-3092

B. Shi https://orcid.org/0000-0003-3306-3764

### Data availability

All of the data that support the findings of this study are available in the main text or Supplementary Information.

## Supplementary materials

Supplementary material 1 Primer information Data type docx Explanation note **table S1**. Amplification primer information and annealed temperature. https://binary.pensoft.net/file/1801589This dataset is made available under the Open Database License (http://opendatacommons.org/licenses/odbl/1.0/). The Open Database License (ODbL) is a license agreement intended to allow users to freely share, modify, and use this Dataset while maintaining this same freedom for others, provided that the original source and author(s) are credited. Zhi Wan Tan, Van Tri Ngo, Boyang Shi

Supplementary material 2 GenBank accession numbers Data type docx Explanation note **table S2**. Species and gene sequences used in the present phylogenetic analysis, with their GenBank accession numbers. https://binary.pensoft.net/file/1801590This dataset is made available under the Open Database License (http://opendatacommons.org/licenses/odbl/1.0/). The Open Database License (ODbL) is a license agreement intended to allow users to freely share, modify, and use this Dataset while maintaining this same freedom for others, provided that the original source and author(s) are credited. Zhi Wan Tan, Van Tri Ngo, Boyang Shi
