# Supplementary material for: A new genus of freshwater crab from Vietnam, with comments on the allied genera Vietorientalia Đặng, 2012, and Hainanpotamon Dai, 1995 (Decapoda, Brachyura, Potamidae)
Source: Zookeys. 2026 Oct 5;1294:61–87. doi: 10.3897/zookeys.1294.199712 (PMC13659257; doi:10.3897/zookeys.1294.199712)
Supplement: Supplementary file 1 [file zookeys-1294-061_article-199712__-s001.docx]

**Supplementary Table S1.** Amplification primer information and annealed temperature.

| **Sequence name** | **Primer (5’-3’)** | **Annealed temperature (°C)** | **Citation** |
| --- | --- | --- | --- |
| 16S | TGAATAACTTGTGGCTGAGCG  AGCTGATAAGGCAGACACTTGA | 50 | Crandall and Fitzpatrick 1996 |
| CO1 | TAAACTTCAGGGTGACCGAAAAATCA  GCACGTCTACAAATCATAAAGATATTGG | 55 | Folmer et al. 1994 |
| 28S | CAGCCCTAAGCAGGTGGTAAACT  CCATCTTTCGGGTCCCAACAT | 52 | Ji et al. 2016  Shi et al. 2024 |
| H3 | ATGGCTCGTACCAAGCAGACVGC  ATATCCTTRGGCATRATRGTGAC | 48 | Colgan et al. 1999 |

**References**

Colgan DJ, McLauchlan A, Wilson GD, Livingston SP, Edgecombe GD, Macaranas J, Cassis G, Gray MR (1999) Histone H3 and U2 snRNA DNA sequences and arthropod molecular evolution. Australian journal of Zoology 46(5): 419–437. https://doi.org/10.1071/ZO98048

Crandall KA, Fitzpatrick JF (1996) Crayfish molecular systematics: using a combination of procedures to estimate phylogeny. Systematic Biology 45: 1–26. https://doi.org/10.1093/sysbio/45.1.1

Folmer O, Black M, Hoeh W, Lutz R, Vrijenhoek R (1994) DNA primers for amplification of mitochondrial cytochrome c oxidase subunit I from diverse metazoan invertebrates. Molecular Marine Biology and Biotechnology 3: 294–299.

Ji YK, Sun YF, Gao W, Chu KL, Wang RC, Zhao Q, Sun HY (2016) Out of the Sichuan Basin: rapid species diversification of the freshwater crabs in *Sinopotamon* (Decapoda: Brachyura: Potamidae) endemic to China. Molecular Phylogenetics and Evolution 100: 80–94. https://doi.org/10.1016/j.ympev.2016.04.003

Shi BY, Pan D, Zhang KQ, Gu TY, Yeo DCJ, Ng PKL, Cumberlidge N, Sun HY (2024) Diversification of freshwater crabs on the sky islands in the Hengduan Mountains Region, China. Molecular Phylogenetics and Evolution 190: 107955. https://doi.org/10.1016/j.ympev.2023.107955
