# Supplementary material for: A new genus of freshwater crab from Vietnam, with comments on the allied genera Vietorientalia Đặng, 2012, and Hainanpotamon Dai, 1995 (Decapoda, Brachyura, Potamidae)
Source: Zookeys. 2026 Oct 5;1294:61–87. doi: 10.3897/zookeys.1294.199712 (PMC13659257; doi:10.3897/zookeys.1294.199712)
Supplement: Supplementary file 2 [file zookeys-1294-061_article-199712__-s002.docx]

**Supplementary Table S2.** Species and gene sequences used in the present phylogenetic analysis, with their GenBank Accession Numbers.

| **Subfamily** | **Species** | **GenBank Accession No.** | | | |
| --- | --- | --- | --- | --- | --- |
|  |  | **16S** | **COI** | **28S** | **H3** |
| Potamiscinae | *Calcipotamon puglabrum* | PP852286 | PP853169 | PZ935270 | PZ946240 |
| Potamiscinae | *Hainanpotamon daiae* | PZ951336 | PZ925147 | PZ935271 | PZ946241 |
| Potamiscinae | *Hainanpotamon orientale* | PP852289 | PP853172 | PZ935272 | PZ946242 |
| Potamiscinae | *Hainanpotamon helense* | PP852288 | PP853171 | PZ935273 | PZ946243 |
| Potamiscinae | *Hainanpotamon fuchengense* | PP852287 | PP853170 | PZ935274 | PZ946244 |
| Potamiscinae | *Lacunipotamon cymatile* | PP852290 | PP853173 | PZ935275 | PZ946245 |
| Potamiscinae | *Lacunipotamon yuanshi* | PP852291 | PP853174 | PZ935276 | PZ946246 |
| Potamiscinae | [*Tiwaripotamon edostilus*](https://www.researchgate.net/figure/Tiwaripotamon-edostilus-Ng-Yeo-2001-A-B-male-CW-420-mm-NCHUZOOL-13613-dorsal_fig7_262693098) | PZ951337 | PZ925148 | PZ935277 | PZ946247 |
| Potamiscinae | *Tiwaripotamon pingguoense* | PP852293 | PZ925149 | PZ935278 | PZ946248 |
| Potamiscinae | *Tiwaripotamon vixuyenense* | PP852294 | PZ934526 | PZ935279 | PZ946249 |
| Potamiscinae | *Tiwaripotamon xiurenense* | PZ951338 | PZ925150 | PZ935280 | PZ946250 |
| Potamiscinae | *Vietorientalia glabra* | PP852295  PP852296 | PP853178  PP853179 | PZ935281  PZ935282 | PZ946251  PZ946252 |
| Potamiscinae | *Dangmon rubrum* gen. nov., comb. nov. | PZ951339  PZ951341 | PZ934527  PZ934528 | PZ935284  PZ935283 | PZ946253  PZ946254 |
| Potaminae | *Lobothelphusa bagoensis* | PP852292 | PP853175 | PZ935285 | PZ946255 |
| Potaminae | *Paratelphusula* *gibbosa* | PZ951340 | PZ925151 | PZ935286 | PZ946255 |
